# Innovative Geopolymer-Based Stabilization of Clayey Soil Using Alkali-Activated Construction and Demolition Wastes: Insights from Large-Scale-Model Tests

**DOI:** 10.3390/polym18212793

**Published:** 2026-11-08

**Authors:** Baki Bağrıaçık, İlkim Özbahçeci Gökdeniz

**Affiliations:** Civil Engineering Department, Faculty of Engineering, Cukurova University, Balcali, Adana 01330, Turkey

**Keywords:** construction demolition wastes, geopolymer, green environment, model test soil improvement, sustainability, waste disposal

## Abstract

In developing and currently developed countries, dealing with solid wastes comprises one of the ultimate social, economical and environmental problems. Thus, geotechnical engineers have recently been in search of alternative waste materials in regard to the utilization of wastes in soil stabilization procedures. Construction demolition wastes, which constitute a significant part in the mentioned solid waste materials, have come to mind as an alternative to be implemented in such applications. By means of considering the utilization of these wastes, one aim has been to reduce both the amount of accumulated waste to the environment and related costs of stabilizing problematic soil conditions. Within the context of this study, improvability of fine-grained soils was questioned by means of utilizing alkaline activators to geopolymerize construction demolition wastes in order to procure an alternative material to cement. Eventually, optimum values for the considered NaOH, KOH and CaO alkaline activators were determined to be 8, 8 and 6 molal, respectively. The use of KOH, NaOH, and CaO activators during the geopolymerization process resulted in a significant improvement in bearing capacity compared with the untreated waste–soil mixtures. This finding indicates that these activators can be effective in enhancing the engineering performance of waste–soil mixtures. Among the activators investigated, NaOH exhibited the highest effectiveness in improving bearing capacity, yielding superior results compared with KOH and CaO. The superior performance of NaOH is considered to be potentially associated with its ability to promote the formation of a binding structure and facilitate interactions among the mixture constituents more effectively during geopolymerization. The resulting binding structure may strengthen the interparticle bonds between the soil and waste particles, thereby contributing to an improvement in the load-bearing capacity of the mixture. Therefore, based on the findings obtained, NaOH was concluded to be the most effective activator for enhancing bearing capacity under the conditions investigated. Additionally, temperature increments resulted in enhancements in the bearing capacities, and the maximum value was reached at the completion of day 28. Moreover, time-dependent settlements reduced by approximately up to 5.76–5.78 times. Consequently, it was observed that the geopolymerization method can be considered as a decent enough and novel alternative to cement for the improvement of engineering properties of fine-grained soils at significant ratios.

## 1. Introduction

Soil improvement methods are generally utilized for the purpose of enhancing the engineering properties of problematic soil conditions. In this aspect, a great deal of procedures and additive materials are put to use. If the relevant literature is assessed, it can be seen that several studies have been performed in the implementation of various additive materials in soil improvement procedures. Some of the mentioned additives can be counted as recycled asphalt pavement (RAP) [1,2], cement by-pass dust (CBPD) or cement kiln dust (CKD) [3,4], pond ash (PA) [5], steel slag (SS) [6,7,8], end-of-life tires (ELT) [9,10,11], fly ash (FA) [12], rice husk ash (RHA) [13,14], waste stone powder (WSP) [15,16], construction and demolition waste (CDW) and other additives. Most of the studies in the relevant literature have pointed out the possibility of successfully utilizing recycled and waste materials in order to improve the engineering properties of soil mixtures and enabling the implementation of decent quality control procedures in the field for the mentioned materials.

In recent years, instead of the above-mentioned traditional additives, geopolymer materials have become popular as a new approach to be taken into consideration in soil improvement applications. However, an overview of the relevant literature brings out the fact that not enough studies have been performed in regard to the implementation of geopolymer binders in the improvement of soils. FA-based geopolymers have been utilized by researchers in order to enhance the engineering properties of clayey soil conditions [17,18,19,20]. On the other hand, within the context of the mentioned studies, the utilized samples were subjected to curing under increasing heat effects or at ambient heating conditions for relatively long time periods, which can be up to a year. The possibility of utilizing a metakaolin-based geopolymer to be a future generation alternative to the currently used soil-stabilizing additive materials was investigated by Zhang et al. [21]. Ground granulated blast furnace slag (GGBS)-based geopolymer binders were experimented on, and it was pointed out that they reached significant degrees of strength in just hours without the need to be subjected to high-temperature curing durations [22,23]. There has not been a comprehensive study in the relevant literature on the impact of the implementation of GGBS-based geopolymers in soil improvement. A study analyzing the impacts of alkali-activating substances on the effectiveness of GGBS-based geopolymers on soft soils is the only exception, which was performed recently by Yaolin et al. [24]. An extensive study has not yet been executed on the analysis of parameters such as the binder content, alkali-to-binder ratio and molar strength of alkali substances, which have impacts on the mechanical properties of the mentioned GGBS-based geopolymers on soils. An Artificial Neural Network (ANN) was developed in order to predict the unconfined compressive strength of clays and to analyze the related parameters [25]. In the study, the behavior of GGBS, FA along with GGBS and FA mixtures (GGBS + FA) was specified in terms of geopolymerization, and the obtained results were discussed.

It was initially pointed out by Davidovits [26] that geopolymers come to existence as the result of a reaction that occurs between a solid alumino-silicate and a high-concentrate aqueous alkali hydroxide or silicate solution. The mentioned alumino-silicate gets subtilized in the alkaline solution and forms a gel-like material. Afterwards, the structure of the system alters by means of the occurrence of increased degrees of bonding ability of the gel network. This process leads to the formation of a three-dimensional alumino-silicate network incorporated with geopolymer materials [27]. It was expressed by Xu and van Deventer [28] that the aforementioned effects are enhanced in cases where the utilized alumino-silicate sources, such as slag, ashes and metakaolin, are subjected to heating. Geopolymers have been taken into consideration within the context of several studies on aspects of civil engineering in regard to improving the engineering properties of cement, mortar and concrete [27,29,30,31,32]. The effects of curing and local soil conditions for the implementation of alkali-activated cements (AACs) on soil improvement procedures have been investigated recently by Obana et al. [33] for marine sediments; Žurinskas et al. [34], Sukmak et al. [35], Cristelo et al. [18,36] and Peirce et al. [37] for clayey soils; and Zhang et al. [21], Rios et al. [38] and Phummiphan et al. [39] on various soil conditions. More detailed studies are still needed for the implementation of such procedures in soil improvement work, which differ vastly compared to the utilization of cement, mortar and concrete, in regard to the applied method and considered soil conditions. Even though considerable degrees of enhancements were encountered in the engineering properties of treated soils within 28–90-day periods [38], initial strength gain and long-term behavior (180 days or more) vary vastly according to the type of the utilized alumino-silicate source, the liquid/solid ratios, along with the type and concentration of alkaline solutions [40]. It was expressed by Cristelo et al. [36,41] that the activator/ash ratio significantly depends on the mechanical strength of the treated mixtures, which is a major factor to be considered in such applications.

Alkali-activated cement (AAC) was utilized as an alternative to Portland cement by Rios et al. [42] in regard to the possibility of implementing a series of waste materials into the content of cement, such as fly ash and blast furnace slag. There exist very limited studies in the relevant literature for the employment of AAC in unpaved road construction in the case of analyzing resistance to wetting and drying (WD) effects at ambient curing temperatures, along with the observation of the long-term mechanical behavior of the materials in question. In a study performed by Rios et al. [42], silty sand was improved by means of the implementation of an AAC which was synthesized from low calcium fly ash and an alkaline solution produced by the usage of sodium silicate and sodium hydroxide. Strength and stiffness values of the samples evolved significantly far beyond the sequel of a 28-day period besides also possessing a decent degree of short-term strength. At the end, in terms of strength, stiffness and resistance to immersion and WD cycles, AAC was found to be compatible with cement in such applications.

A geopolymer binder was utilized in soil samples at 20, 30 and 40% by Davies [43]. In addition, activated fly ash was used in soil stabilization by Cristelo et al. [36] at the same additive percentages. By means of implementing jet grouting and mechanical mixing procedures, soil improvement aspects of metakaolin- and fly ash-based geopolymers have been examined by researchers, and promising results were achieved [18,21]. It was expressed by Zhang et al. [44] that expansion and heaving events that occur in sulfate-rich soils should be taken into consideration in detail for necessitating long-term observation in the case of improving such soils by means of utilizing calcium-based improvement agents, such as cement and lime, as a result of the interactions taking place between calcium and water. In the study, as alternative stabilizers, calcium-based geopolymers were successfully replaced by the mentioned stabilizing materials. The results indicate that in terms of strength, strain and swelling potential, the metakaolin-based calcium-free geopolymer (MKG) exhibited rewarding improvements in clayey soil conditions. Therefore, focusing on the implementation of higher concentrations of geopolymers along with the geopolymers obtained from some industrial wastes should be taken into consideration on account of providing the aforementioned properties and advantages, especially in cases where sulfate-rich soils are in question.

The literature contains numerous studies investigating the use of alternative materials as substitutes for cement and other conventional binder-based construction materials to mitigate a range of geotechnical problems, including excessive soil settlement, inadequate bearing capacity, seepage, and slope instability. These alternative stabilization approaches primarily aim to reduce the environmental impacts associated with conventional cement-based practices, particularly carbon dioxide (CO_2_) emissions, while simultaneously enhancing the sustainability and engineering performance of geotechnical applications [45,46,47,48,49,50,51,52,53,54,55].

A review of the existing literature indicates that soil stabilization studies have been conducted using geopolymers produced from various waste materials. However, the parameters investigated in these studies have generally been limited in scope, and experimental investigations have predominantly been conducted on a small scale. Furthermore, studies have investigated the production of geopolymers using construction and demolition waste (CDW) with a single activator. An examination of these studies reveals that the investigations have primarily focused on individual performance parameters, such as settlement, hydraulic conductivity, or bearing capacity. Moreover, these studies have generally involved small-scale experiments and have been limited in terms of the range of parameters investigated and the duration of the experimental programs. In contrast, the present study provides a comprehensive and detailed comparative investigation of the effects of different activators on the immediate and time-dependent bearing capacity, settlement, and hydraulic conductivity of geopolymer-stabilized soil. In addition, the study evaluates the potential of these geopolymer-based materials as alternative binders to conventional cement. By considering multiple engineering performance parameters and their temporal variations, this study aims to address the limitations of previous research and to provide a more comprehensive assessment of the applicability of CDW-based geopolymers in soil stabilization.

## 2. Materials and Methods

### 2.1. Materials

In order to obtain the aimed soil type, clayey soil zones were examined at the initial stages of the conducted study. Required amounts of clayey soil samples were collected from Adana Province, which is located in southern Turkey. In the sequel of bringing the soil samples to the soil mechanics laboratory at Çukurova University, they were prepared to be subjected to a series of experiments at first to specify the index properties. By means of the performed specific gravity tests on the clayey soil samples, the unit weight of grains was found to be 27 kN/m^3^. Next, the values of maximum dry unit weight and optimum water content were acquired as 17.40 kN/m^3^ and 18%, respectively. As for the Atterberg Limits, liquid limit, plastic limit and the plasticity index were obtained as 42%, 24% and 18%, respectively, on average.

Soil classification was performed based on the Unified Soil Classification System (USCS) specified in the ASTM D2487 [56] standard. Based on the evaluations, the physical and index properties of the investigated soil indicated that it belongs to the medium-plasticity clay category. This classification provides a fundamental reference for evaluating the engineering behavior of the soil and interpreting its performance following geopolymerization applications. Afterwards, construction demolition wastes, which were later on implemented in alkaline activation processes, were obtained from the ruins of a structure demolished within the scope of an urban renewal project in Adana Province, Turkey. The collected construction demolition wastes included marble, concrete materials such as aggregate and cement along with plaster. With the aid of a specially manufactured plastic tip hammer, the above-mentioned wastes were pulverized into smaller particles. The newly obtained particles were sieved through a 2 mm-sized screen, and the parts that passed under the screen were utilized in the experiments. 

**Figure 1:**
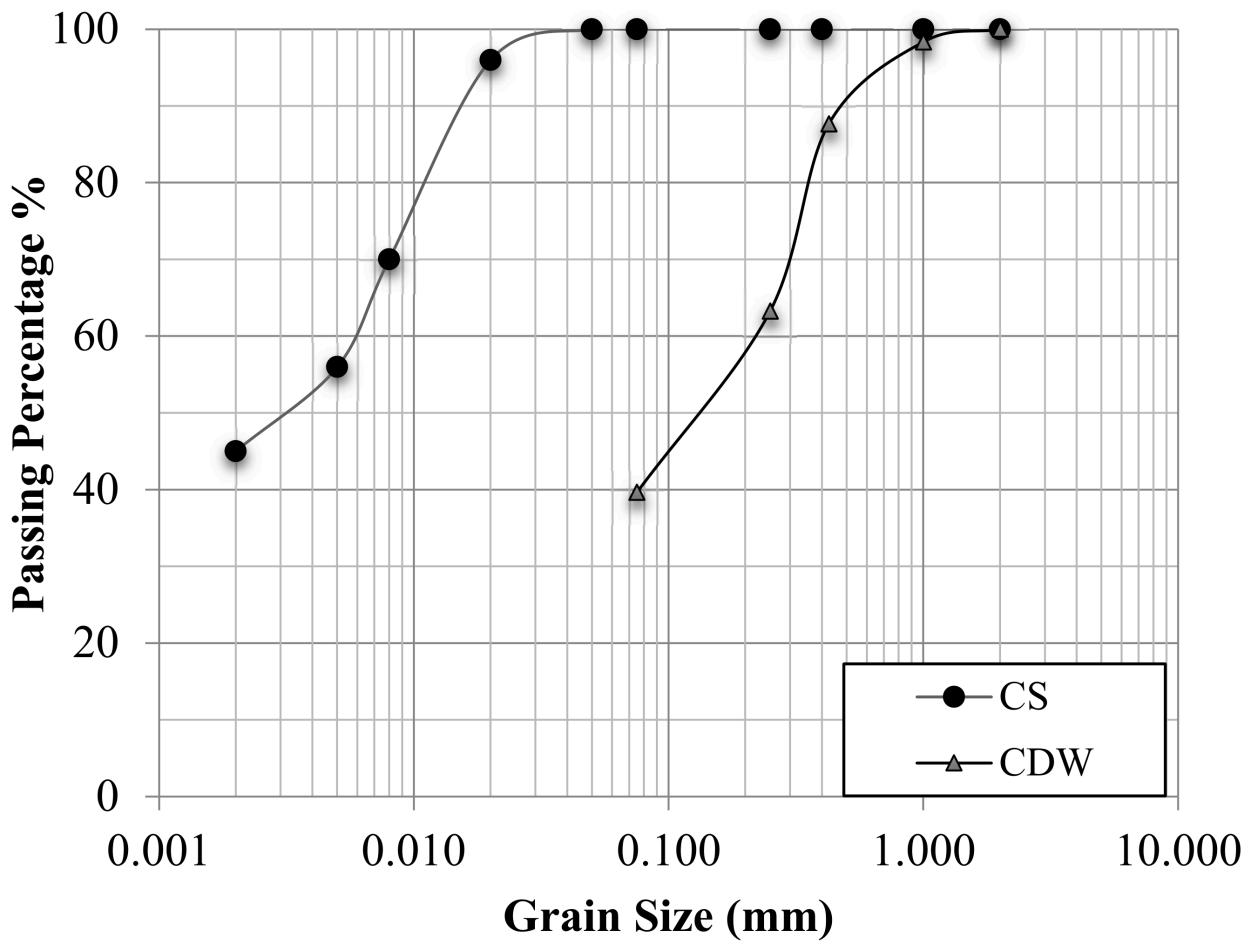
Sieve analysis results for CS and CDW.

In this study, construction demolition wastes were taken into consideration and implemented in clayey soil samples solely and/or with an alkaline activator agent in order to form monolithic geopolymer materials. A recycling or processing procedure was not carried out for the wastes within the context of the performed study. Wastes were just pulverized into a nearly sand-sized particle size, and parts finer than the 2 mm sieve size were put to use in the experiments. Sieve analysis tests were conducted for both clayey soil samples and pulverized construction demolition wastes, and the obtained results are presented in Figure 1.

In addition, optical microscope and X-ray diffraction (XRD) methods were executed on the pulverized construction demolition wastes and clayey soil samples in order to obtain their chemical structures. The results of the chemical analyses are given in Table 1 below.

It is an obvious fact that the utilization of various alkaline materials results in increases in the cost of the related procedures, along with causing more complex and risky mixing processes [57]. In this regard, sodium hydroxide (NaOH) was implemented as the liquid alkaline activator since it is relatively more compatible with soil improvement criteria and economically favorable. The caustic NaOH solution was supplied from MERCK Chemical Plant in pellet form. The mentioned alkaline activators were diluted in rectified water in order to reach the required concentration states (4 M, 6 M, 8 M, and 10 M).

### 2.2. Methods

Hydraulic reactions are catalyzed by the OH ions present in the NaOH solution in the course of alkali activation stages. The mentioned process results in increments in the pH of the solution. Dissolution and condensation reactions take place between Si and Al minerals existing in the powder material (CW), which is the source of alumino-silicate [27].

#### 2.2.1. Specification of the Mixtures

Potassium hydroxide (KOH), sodium hydroxide (NaOH) and calcium oxide (CaO), in pellet form, were prepared by means of dissolving in pure water at a specific rate and kept at room temperature for a 24 h period in advance of being utilized in the conducted experiments. In the course of preparing the mortar, the powder binding material, which is to be activated, was mixed with the KOH, NaOH and CaO solutions (4 M, 6 M, 8 M, and 10 M) in a Hobart mixer consistently for 3 min. Next, the clayey soil sample was added to the mixture, and the mortar preparation was concluded by means of performing a mixing process for 3 min once again. Later on, the prepared samples, of which the ratio of water/binder was selected as 0.35, were subjected to curing for 7-day periods at ambient temperature (25 °C).

For the preparation of sodium hydroxide (NaOH; molar mass: 40.00 g/mol) solutions, the required amounts of NaOH for concentrations of 4 M, 6 M, 8 M, and 12 M were calculated as 160 g, 240 g, 320 g, and 480 g, respectively. For potassium hydroxide (KOH; molar mass: 56.11 g/mol), the required amounts at the same molarities were determined to be 224.44 g, 336.66 g, 448.88 g, and 673.32 g, respectively. For calcium oxide (CaO; molar mass: 56.08 g/mol), the corresponding amounts were calculated as 224.32 g, 336.48 g, 448.64 g, and 672.96 g, respectively. During the preparation of the NaOH, KOH, and CaO mixtures, the calculated quantities of the solid chemicals were carefully introduced into an appropriate initial volume of distilled water under controlled conditions. After the heat generated during the dissolution or reaction process had dissipated and the mixture had cooled to room temperature, distilled water was added to adjust the final volume to 1 L. This approach was intended to ensure accurate adjustment of the solution molarity.

#### 2.2.2. Preparation of the Soil Samples

In this study, mixtures containing construction and demolition waste (CDW) at proportions ranging from 0% to 20% were prepared to determine the optimum CDW content. Initially, the optimum water content of each mixture was determined. To systematically evaluate the effect of additive dosage on the geotechnical performance of the stabilized soil, the CDW content was increased in increments of 2%. These increments were selected to facilitate the assessment of changes in strength and deformation behavior across the investigated dosage range. Unlike recycled aggregate concrete, in which recycled aggregates can replace a substantial proportion of natural aggregates, the present study focuses on the function of alkali-activated CDW as a soil stabilization material. Therefore, the dosage levels were selected in accordance with the objectives and requirements of geotechnical soil improvement. Subsequently, unconfined compressive strength tests were performed on specimens prepared at their respective optimum water contents to identify the optimum mixture proportions (Table 2). Following the determination of the optimum proportions, the specimens were prepared for large-scale laboratory model tests. All specimen preparation procedures were conducted in accordance with the relevant standard procedures to ensure the homogeneity and consistency of the mixtures.

To activate the construction and demolition waste, alkaline solutions, including potassium hydroxide (KOH), sodium hydroxide (NaOH) and calcium oxide (CaO), were used. Since the dissolution of these chemicals in water generates heat and increases the solution temperature, the alkaline solutions at concentrations of 4 M, 6 M, 8 M, and 10 M were prepared one day before use (Table 3). After the clay and CDW were mixed at their predetermined optimum proportions, the alkaline solution was added, and the mixture was thoroughly blended until a homogeneous consistency was achieved. Upon completion of these procedures, the specimens were ready for testing [27].

Large-scale model tests were conducted using a square model testing box with dimensions of 600 mm × 600 mm × 600 mm. The frame of the model box was constructed from 5 mm-thick steel profiles, while 10 mm-thick glass panels were installed on the front and rear faces to facilitate visual observation of the specimens during placement. The bottom and side surfaces of the model box were constructed from 20 mm-thick wooden panels. A circular steel plate with a diameter of 100 mm and a thickness of 10 mm was used as the model footing.

The soil specimens were compacted using a specially designed drop hammer to ensure consistent compaction energy [58]. The hammer had a mass of 2 kg and was mounted on a system that maintained a constant drop height of 20 cm. The clayey soil was placed in the model box in successive layers, each 2.5 cm thick. Approximately 80 hammer blows were applied to each layer during compaction.

To maintain consistent testing conditions, the water content, surface levelness, and uniformity of each layer were carefully checked during specimen preparation. The placement procedure and the model testing apparatus were also inspected to ensure proper specimen preparation and reliable experimental results.

Before loading commenced, the model footing was positioned centrally on the prepared soil surface to ensure alignment with the center of the model box. Loads and corresponding vertical displacements were measured using an electronic load cell manufactured by ESIT International United Kingdom and electronic vertical displacement transducers manufactured by ELE International United Kingdom, respectively. Two displacement transducers were installed at symmetrically located points on the model footing to measure vertical settlement. The settlement values used to construct the load–settlement curves were calculated as the average of the two displacement measurements.

The load was applied vertically and statically to the model footing under uniform loading conditions. The load cell and displacement transducers transmitted their respective measurements to an EL27-1487 Autonomous Data Acquisition Unit (ADU) United Kingdom equipped with 32 input channels. The recorded data were subsequently processed using the DS7 geotechnical software to obtain numerical load and displacement values. Upon completion of each test, vertical load–settlement curves were constructed, and the corresponding bearing capacities were determined.

The dimensions of the model testing box were selected to minimize potential boundary effects on the experimental results. To verify the adequacy of these dimensions, stress transducers were initially installed at appropriate locations within the model box. The model footing was then loaded, and the stresses developed near its edges were monitored. The measured stress values at the monitored locations were zero, indicating that no detectable boundary effects occurred under the tested conditions. Therefore, the selected model box dimensions were considered adequate for the model tests.

## 3. Results

### 3.1. Effects of Water Content and Mixing Ratio

At this stage of the conducted study, compaction tests were carried out in order to determine the optimum ratio of construction demolition wastes to be implemented in the mixtures and the required water content values for the mixtures prepared at optimum ratios. In regard to the performed test results, the optimum waste ratio was found to be 14%. In the case where construction demolition wastes were implemented in the mixtures at a 14% ratio, optimum water content and maximum dry unit weight were obtained as 17.05% and 18.03 kN/m^3^, respectively.

**Figure 2:**
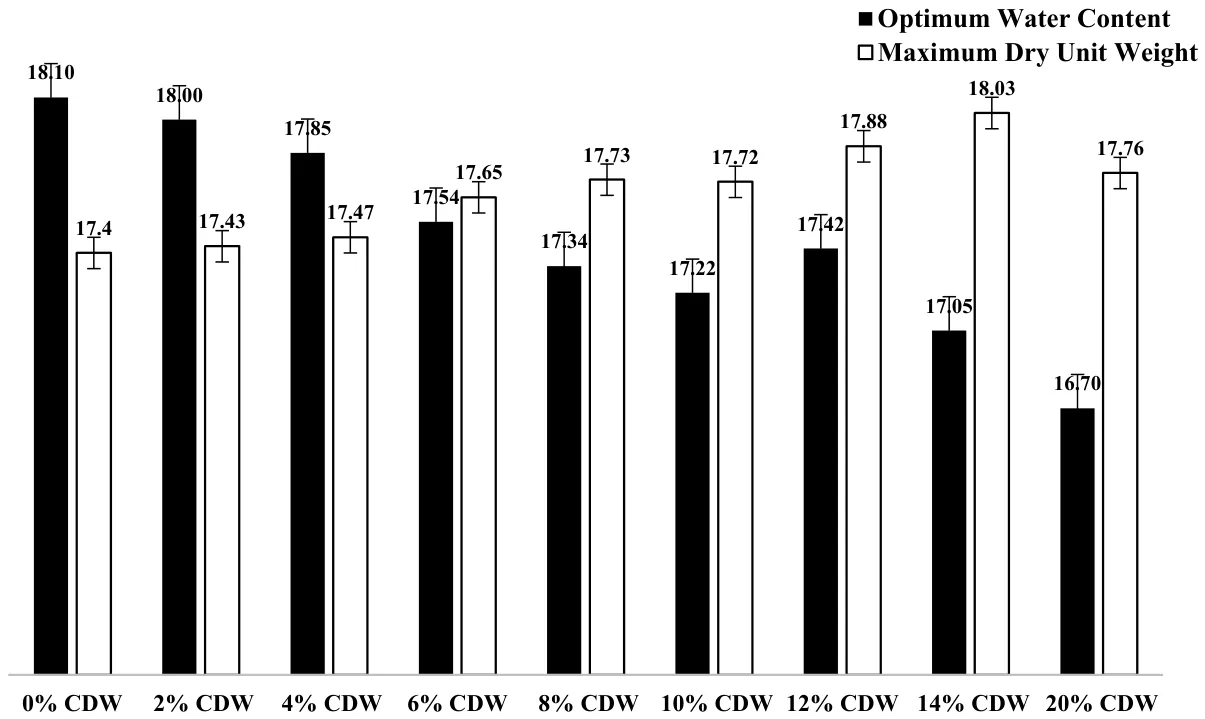
The values of optimum water content and dry unit weight with respect to various CDW ratios.

In consequence with the acquired results, in the course of carrying out the large-scale laboratory model experiments, the optimum construction demolition waste ratio was utilized as 14%, whereas the optimum water content was adjusted to be 17.05%. The above-mentioned relations are given in Figure 2 below.

### 3.2. Bearing Capacity of the Clayey Soil

**Figure 3:**
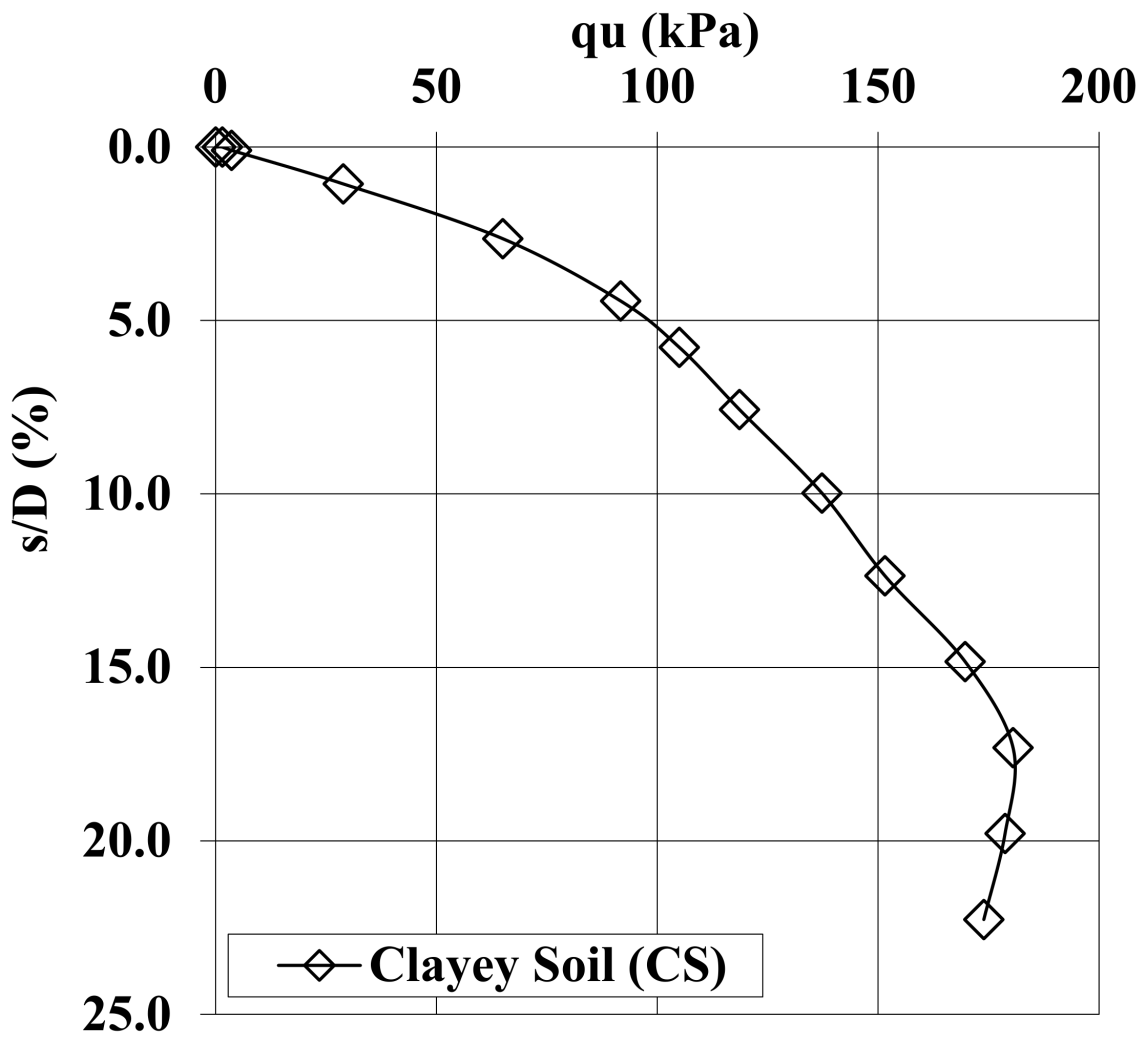
Load–displacement curve for the clayey soil.

In Figure 3, the bearing capacity of the clayey soil samples prepared at optimum water content can be observed. In compliance with the outcome of the performed large-scale laboratory model experiments, the bearing capacity value for a foundation to be built on such soils was determined to be 180.532 kPa. For the cases where structures are planned to be built on such soil conditions, soil improvement applications may be a necessity as a result of inadequate degrees of bearing capacities of the soils at hand.

### 3.3. Molarity Effect

In the study, KOH, NaOH and CaO alkaline activators in various molarities were utilized in order to activate the implemented construction demolition wastes. The acquired outcomes are commented on thoroughly below.

#### 3.3.1. KOH Alkaline Activator

**Figure 4:**
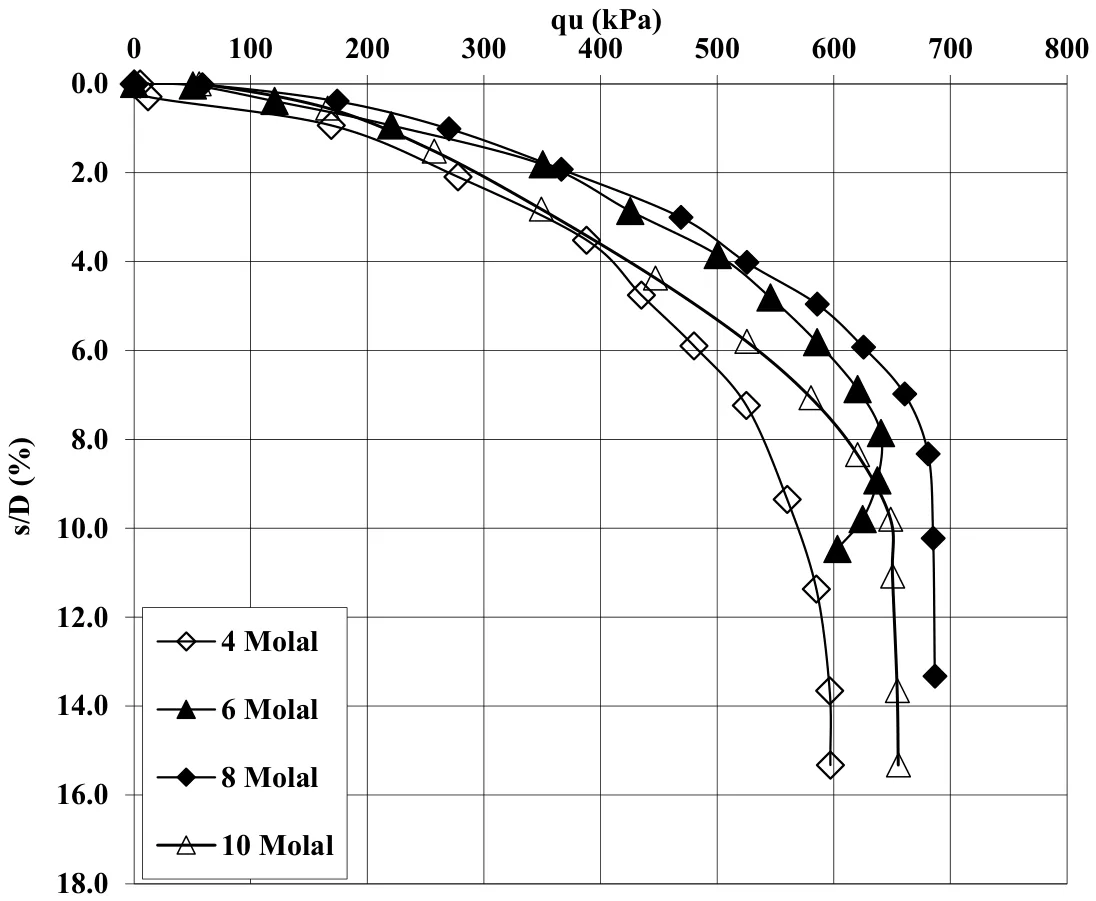
Load–displacement curves for various molarities of KOH.

**Figure 5:**
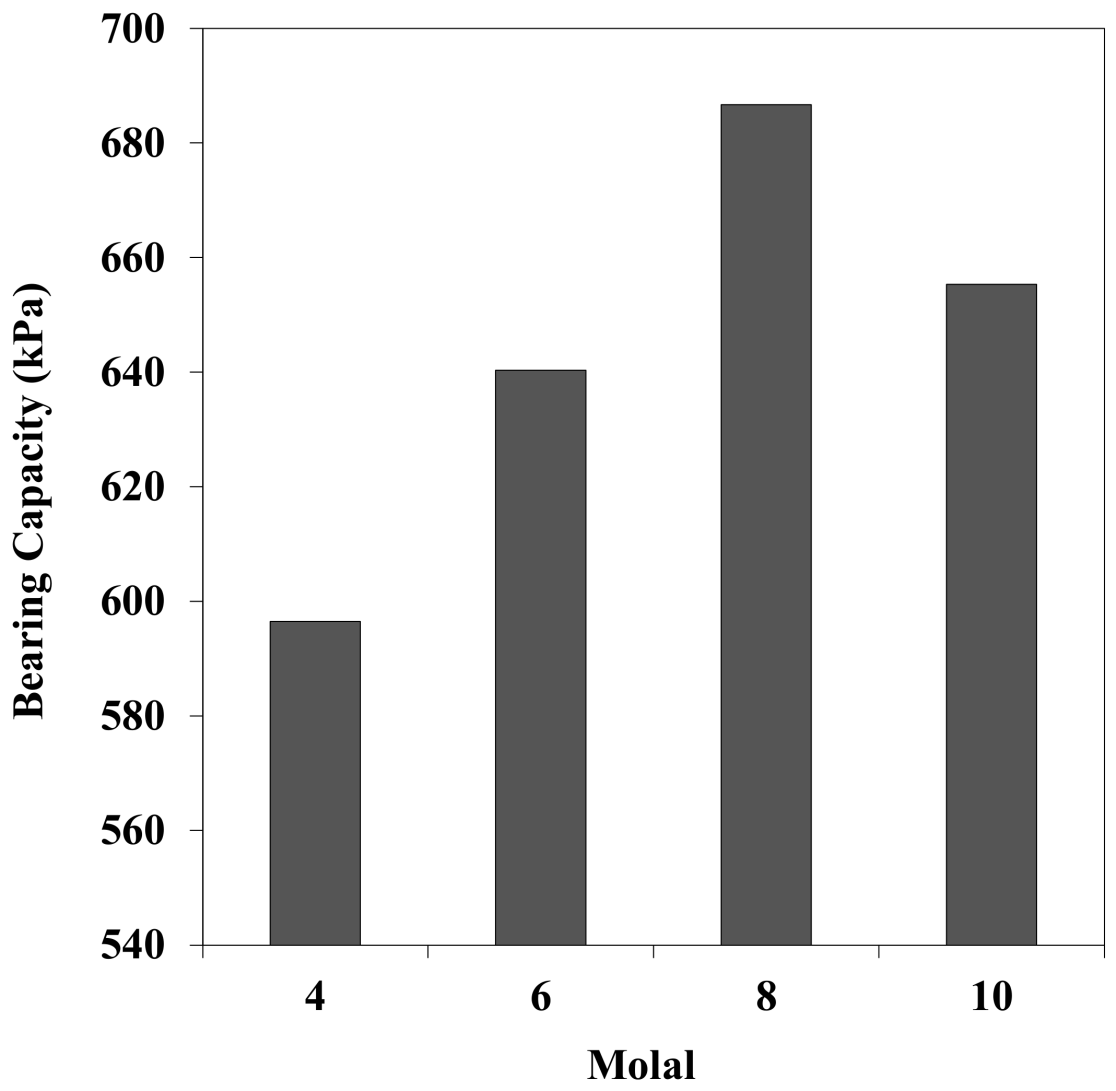
Bearing capacity values for various molarities of KOH.

In Figure 4 and Figure 5 below, the results for experiments which were performed for the case where KOH was used as an alkaline activator in various molarities can be seen. The bearing capacity values for the mixtures of clayey soil samples and construction demolition wastes activated with the utilization of KOH solutions in 4, 6, 8 and 10 molal were determined to be 585.48 kPa, 640.33 kPa, 680.83 kPa and 648.65 kPa, respectively. Thus, it was stated that significant improvement degrees were attained for all of the utilized molarity values in comparison to the case where just clayey soil samples were implemented. On the other hand, the highest bearing capacity was achieved for the molarity value of 8. Hence, it was advised that an optimum molarity value of 8 should be considered in the activation processes of clay-construction demolition waste mixtures by means of utilizing KOH activators.

If the relevant literature is assessed, it can be seen that no study considering the activation of construction demolition wastes in order to produce geopolymers is present. However, some relevant studies in the literature considered the improvement of soils by means of using KOH activators in different waste materials. KOH was used by Alsafi et al. [59] to activate fly ash, where the optimum ratio was specified as 12 molal out of the utilized molarities of 8, 10 and 12. In this study, for the KOH activator, the optimum ratio was determined to be 8 molal, which is a more economical outcome.

#### 3.3.2. NaOH Alkaline Activator

**Figure 6:**
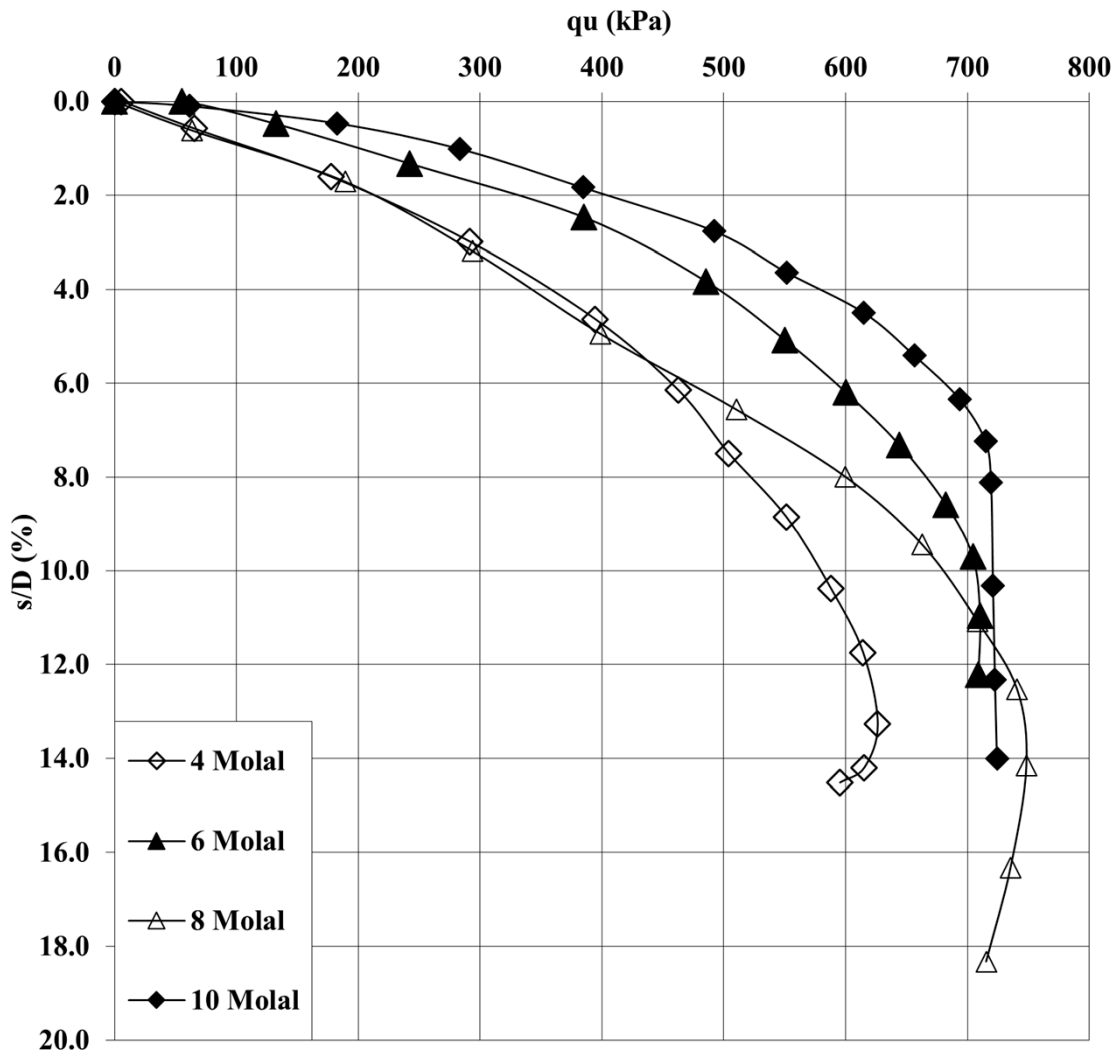
Load–displacement curves for various molarities of NaOH.

**Figure 7:**
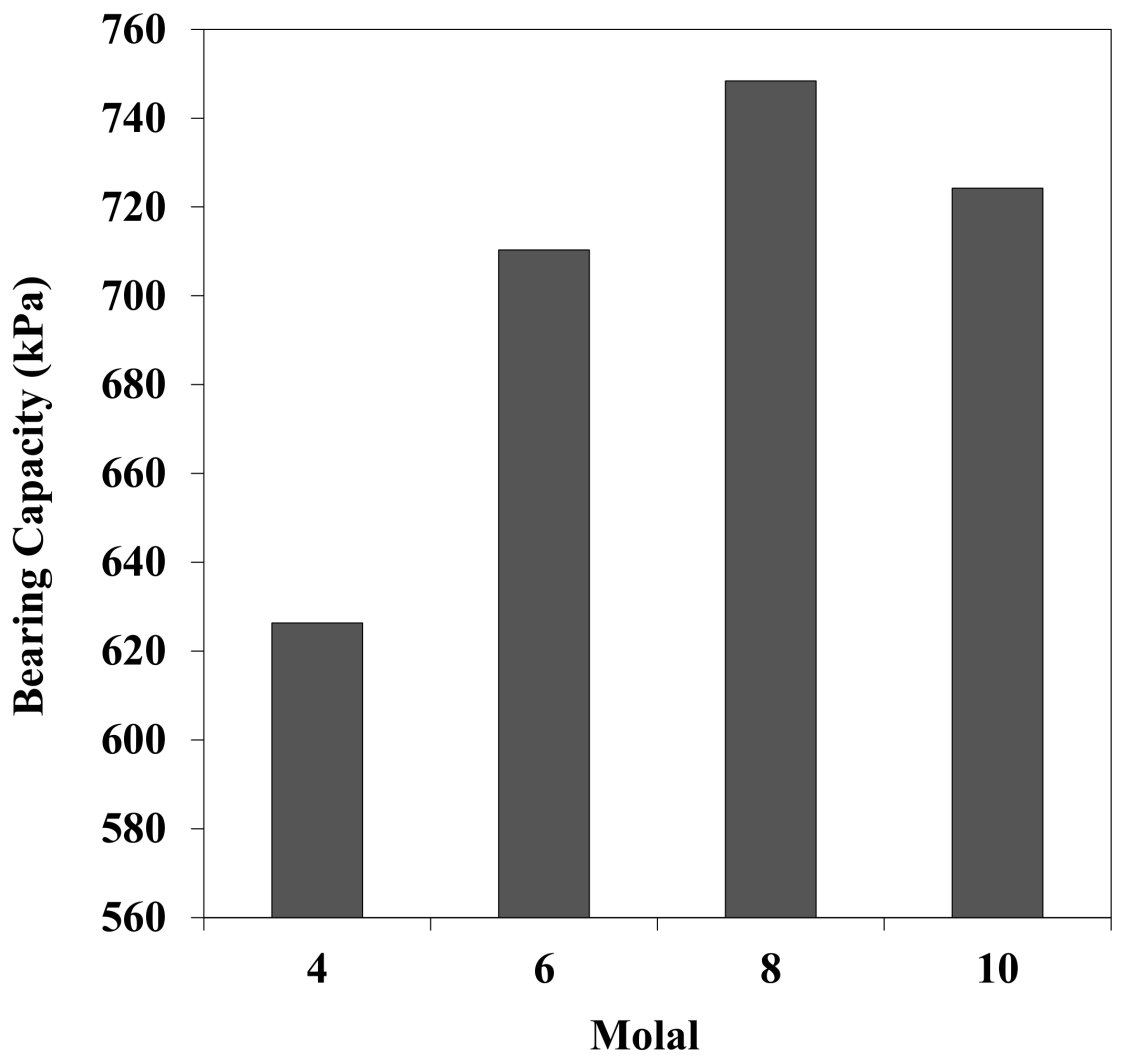
Bearing capacity values for various molarities of NaOH.

In Figure 6 and Figure 7, the results of the experiments implementing the NaOH alkaline activator in various molarities are given. The bearing capacities for the case at hand were found to be 626.30 kPa, 708.41 kPa, 748.37 kPa and 719.37 kPa for the molarity values of 4, 6, 8 and 10, respectively. Once again, considerable degrees of improvements were acquired for each molarity value compared to the implementation of just clay. As in the case of using a KOH activator, the highest bearing capacity was determined in NaOH activator utilization as 8 molal, as well. Therefore, 8 molal was once again chosen to be the optimum alkaline activator ratio for NaOH.

NaOH was implemented as an alkaline activator in 8, 10 and 12 molal in a study performed by Alsafi et al. [59] within the context of which the optimum ratio was found to be 12. Once again, the obtained 8 molal value for the optimum activator ratio in this study stands out as a more economical solution for such procedures.

#### 3.3.3. CaO Alkaline Activator

**Figure 8:**
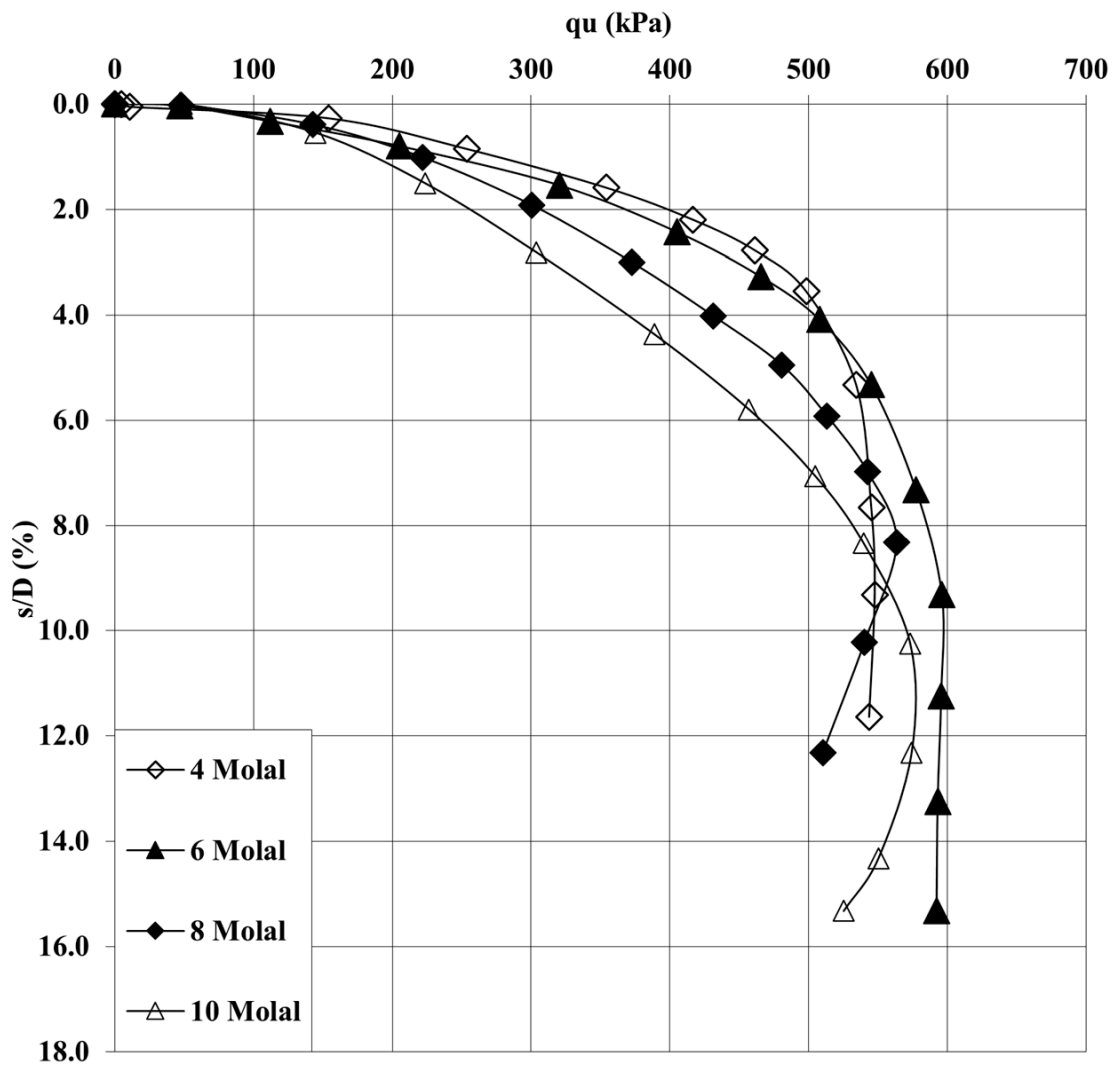
Load–displacement curves for various molarities of CaO.

**Figure 9:**
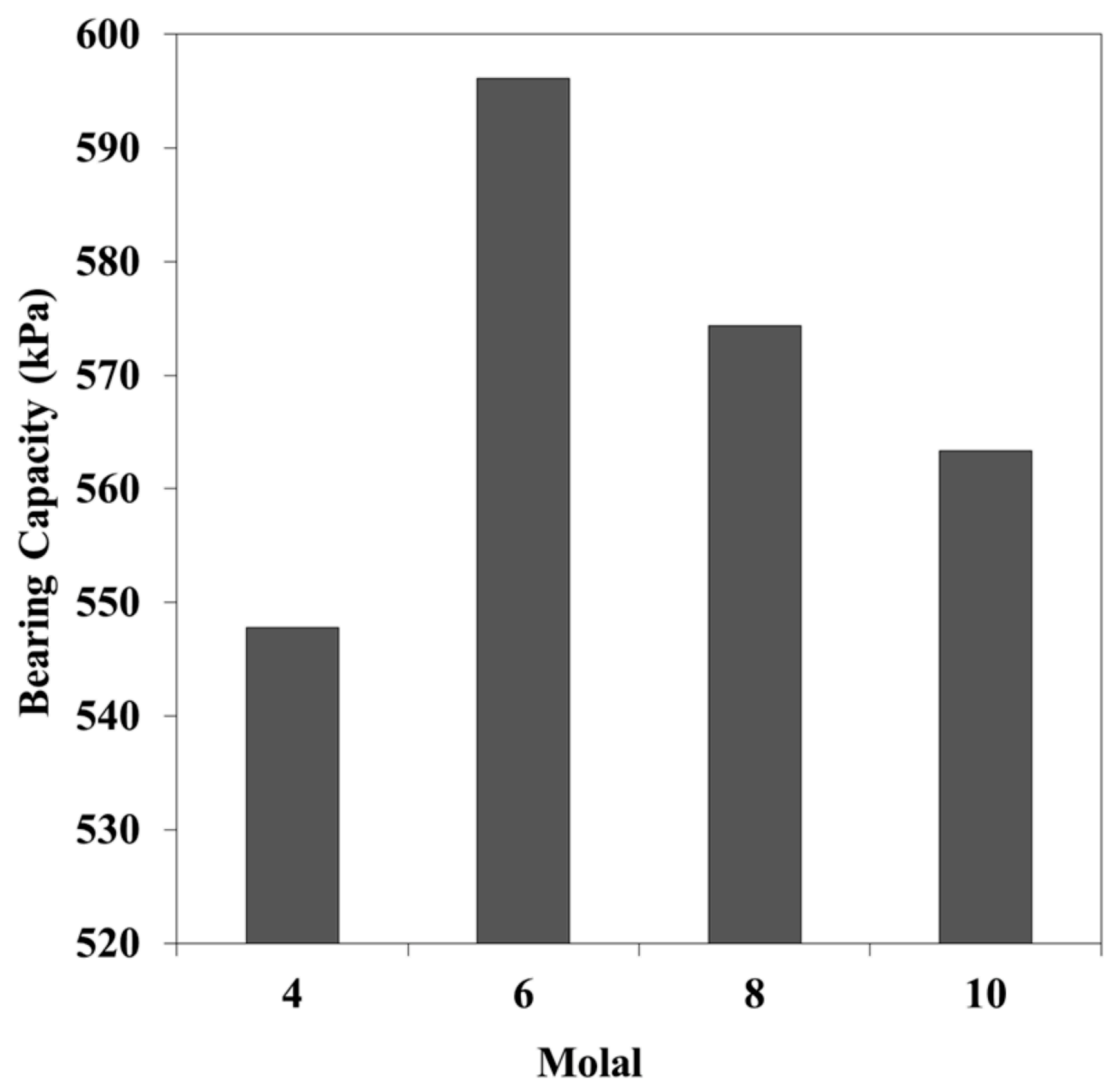
Bearing capacity values for various molarities of CaO.

The conducted experiment results for the cases implementing the CaO alkaline activator in various molarities are given in Figure 8 and Figure 9 below. The bearing capacity values for the mixtures activated with CaO solutions in 4, 6, 8 and 10 molal were obtained as 545.36 kPa, 596.14 kPa, 563.33 kPa and 573.33 kPa, respectively. Significant improvements were recorded for all of the utilized molarities in comparison to the case where neat clay was used. The highest degree of bearing capacity was attained for 6 molal with Cao alkaline activator utilization, which was chosen to be the optimum value. Examining the relevant literature, no study which includes the utilization of CaO as an alkaline activator in order to activate construction demolition wastes has been observed.

### 3.4. The Effect of Alkaline Activator

**Figure 10:**
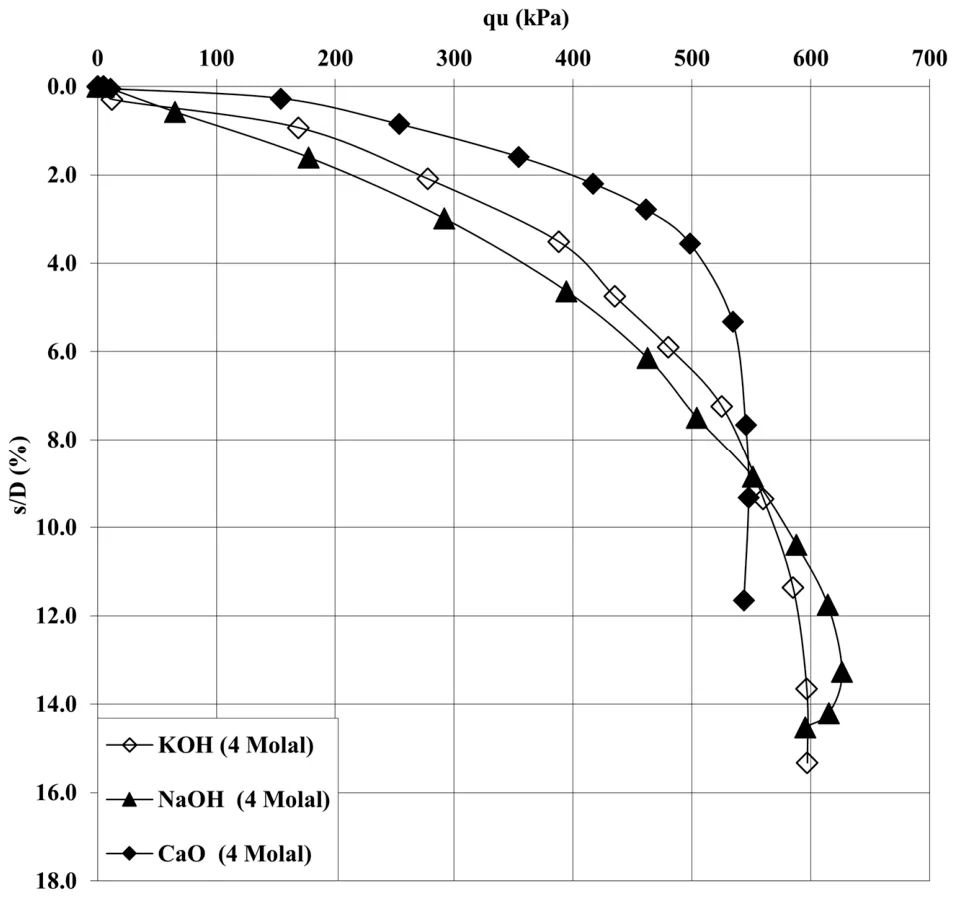
Load–displacement curves for various activators for a molarity value of 4.

**Figure 11:**
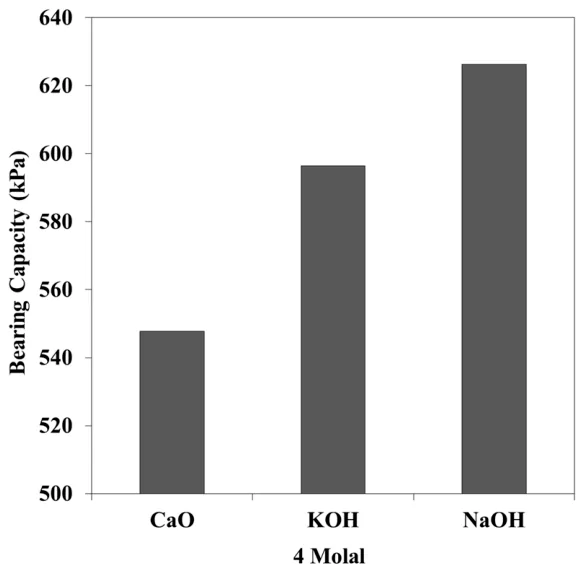
Bearing capacity values for various activators for a molarity value of 4.

**Figure 12:**
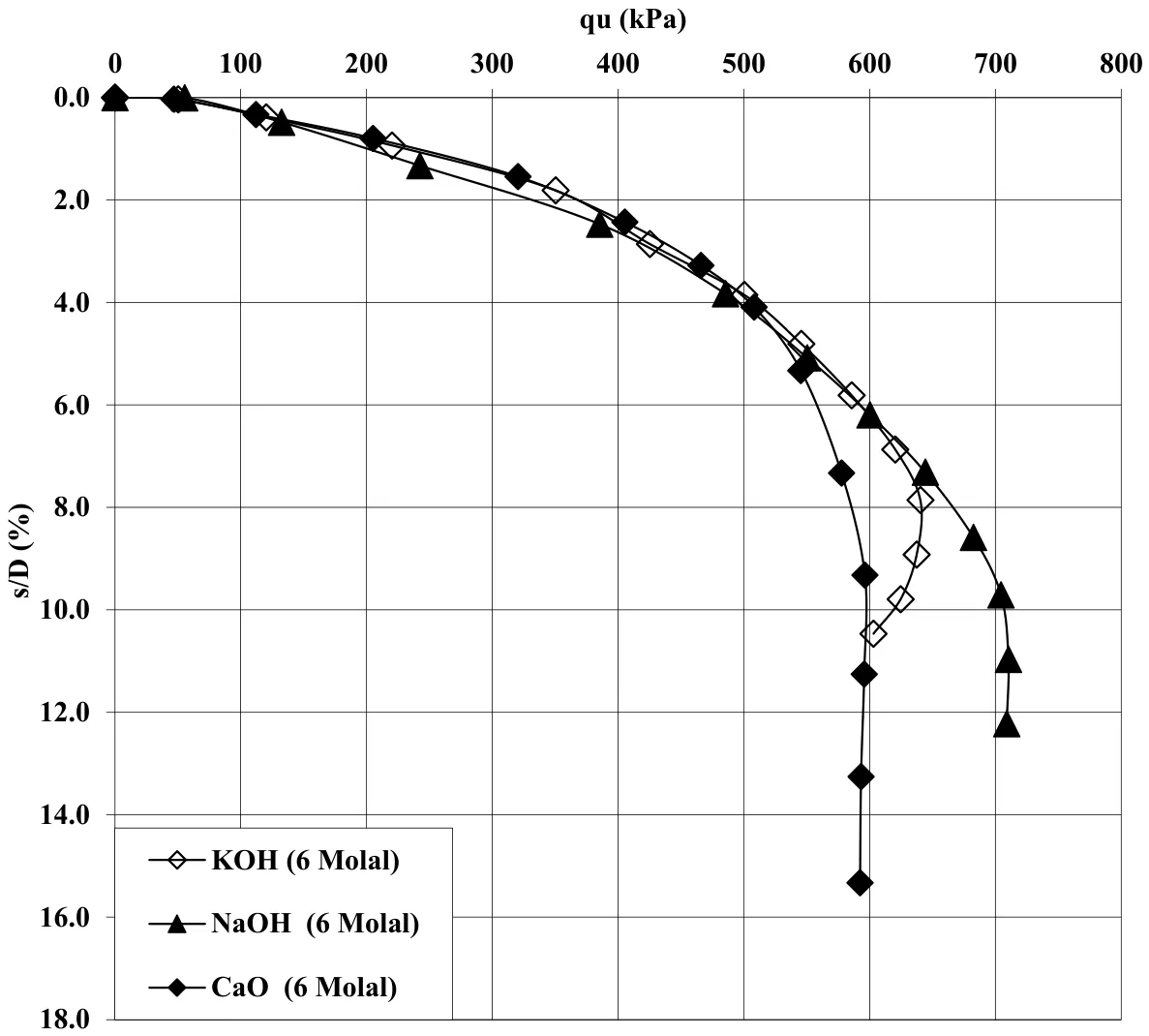
Load–displacement curves for various activators for a molarity value of 6.

**Figure 13:**
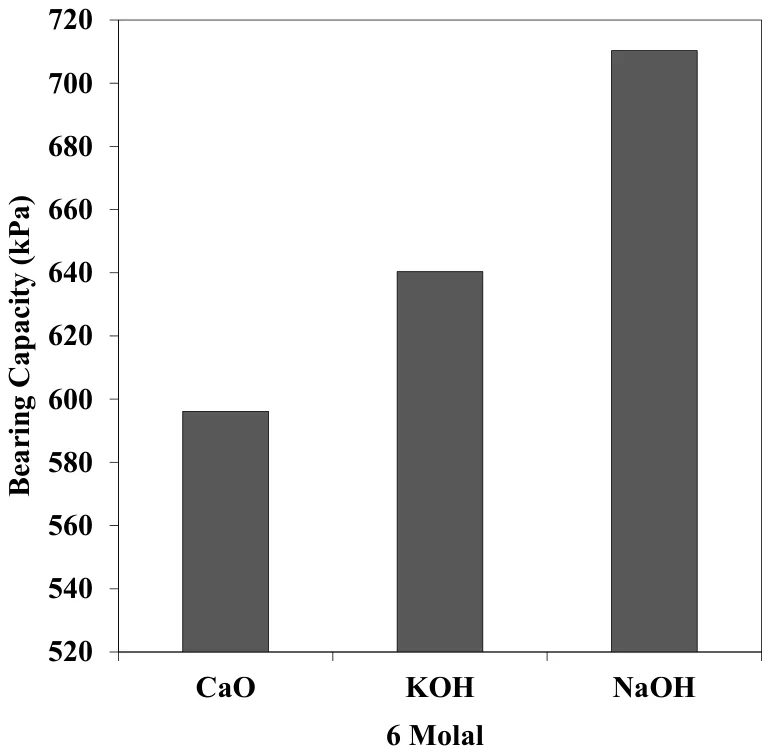
Bearing capacity values for various activators for a molarity value of 6.

**Figure 14:**
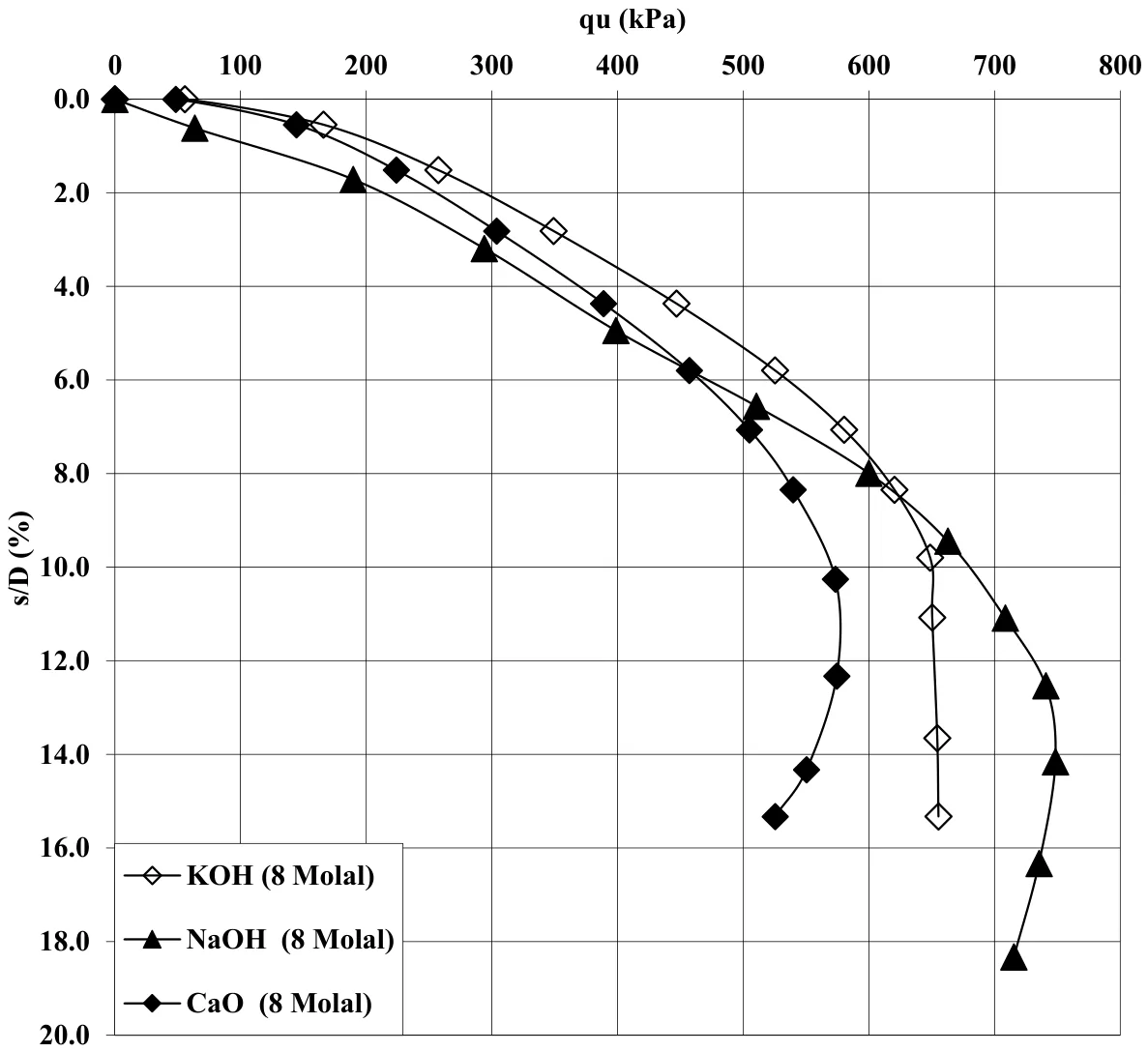
Load–displacement curves for various activators for a molarity value of 8.

**Figure 15:**
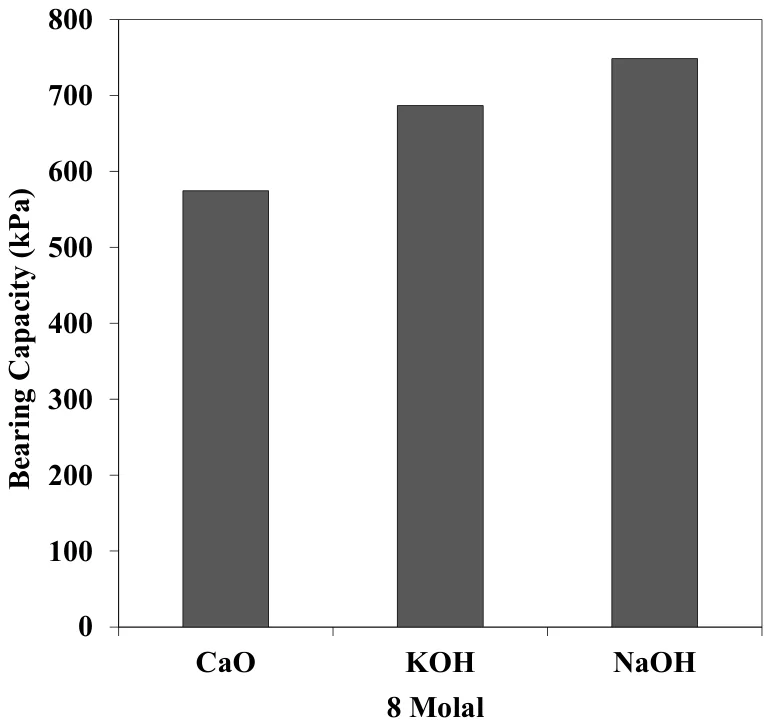
Bearing capacity values for various activators for a molarity value of 8.

**Figure 16:**
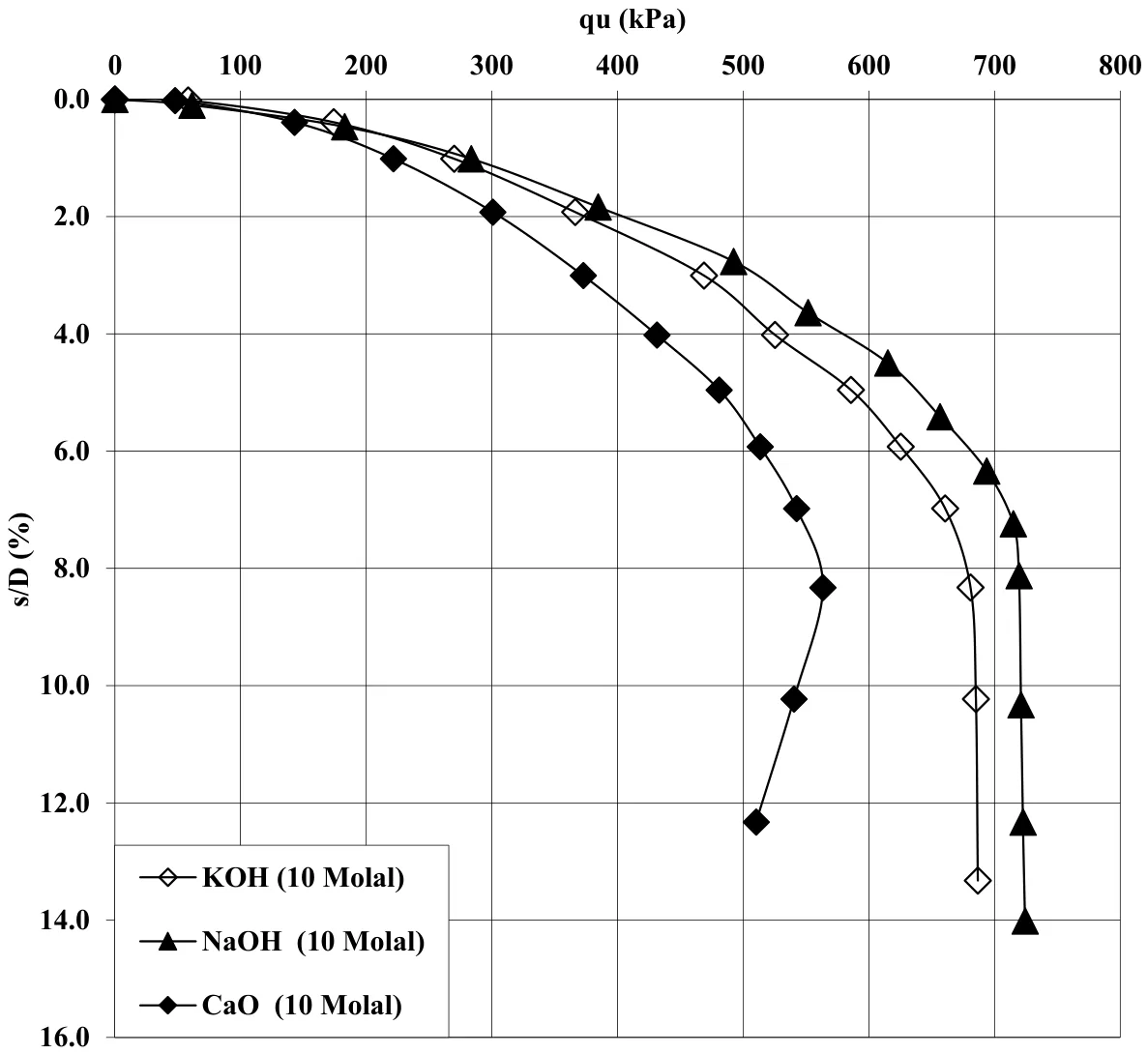
Load–displacement curves for various activators for a molarity value of 10.

**Figure 17:**
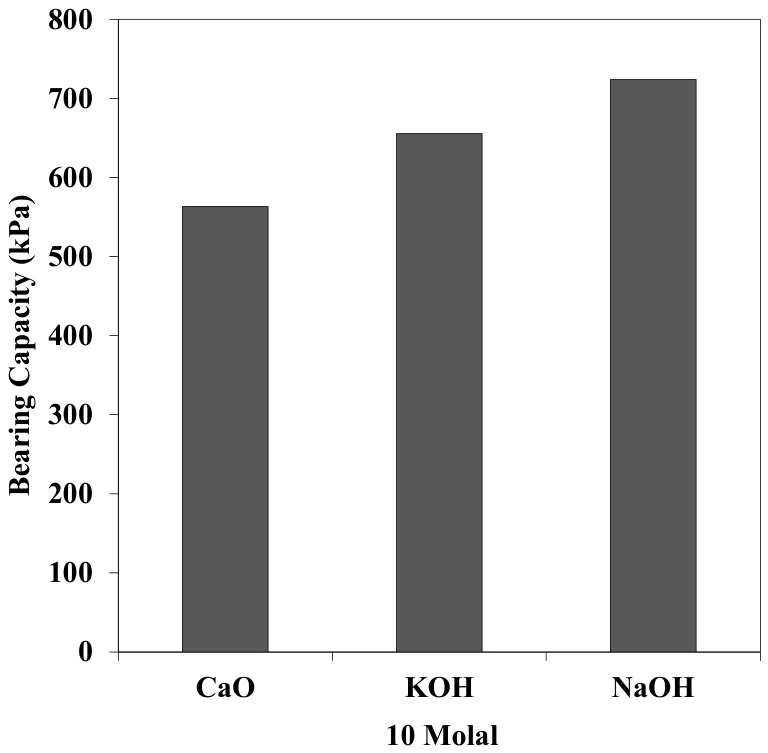
Bearing capacity values for various activators for a molarity value of 10.

In Figure 10, Figure 11, Figure 12, Figure 13, Figure 14, Figure 15, Figure 16 and Figure 17, the conducted comparisons can be observed for different alkaline activators with respect to bearing capacities. In regard to the obtained results, as an activator to be implemented in the mixtures of clay and construction demolition wastes, NaOH presented the highest improvement ratios out of the three kinds of alkaline activators for all of the considered molarity values. The reason for this phenomenon was thought to be the occurrence of higher degrees of reactions that took place amongst the components of the NaOH alkaline activator and construction demolition wastes in comparison to the cases for KOH and CaO utilization. Therefore, the implementation of NaOH alkaline activators is advised to be the preferred case in such improvement conditions.

### 3.5. The Effect of Curing Temperatures

**Figure 18:**
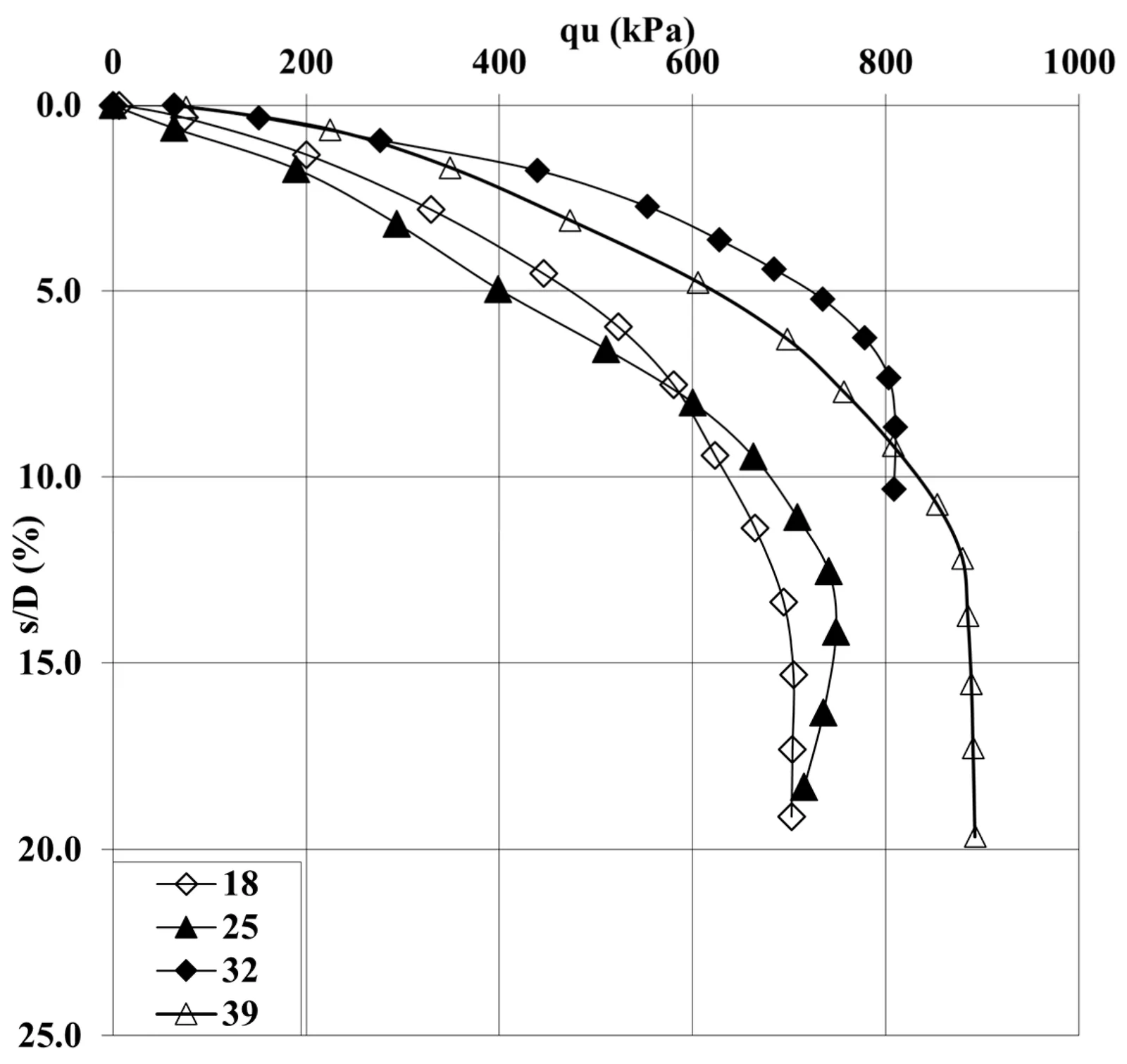
Load–displacement curves for various curing temperatures (degrees Celsius).

**Figure 19:**
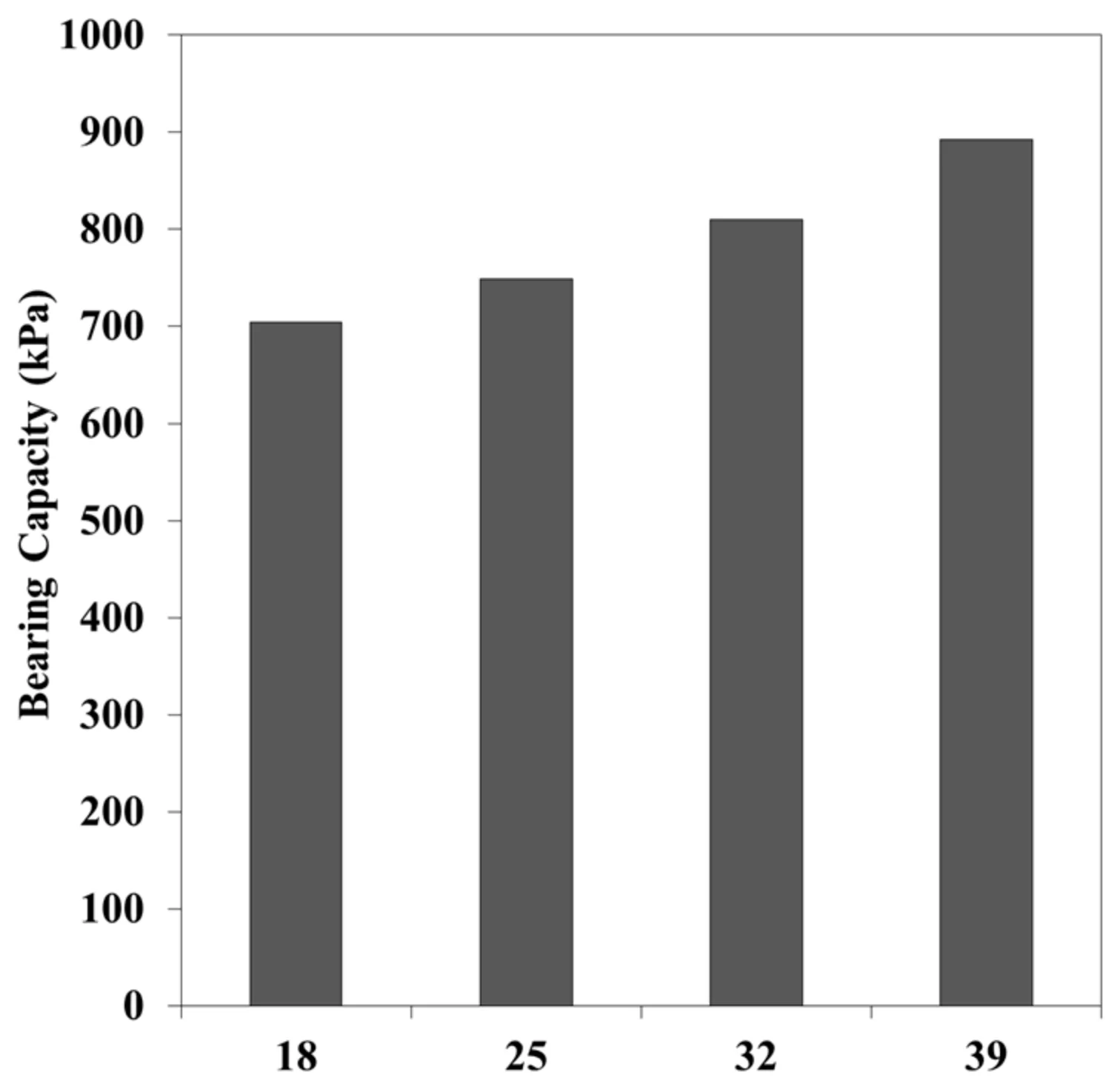
Bearing capacity values for various curing temperatures (degrees Celsius).

At this stage of the study, the mixture samples prepared at optimum ratios and by means of activating with the most ideal activator conditions were subjected to curing under temperatures of 18 °C, 25 °C, 32 °C and 39 °C. The corresponding relationships can be seen in Figure 18 and Figure 19 below. In accordance with the arrived results, the bearing capacity values were determined to be 704.33 kPa, 748.37 kPa, 802.97 kPa and 888.65 kPa for the curing temperatures of 18 °C, 25 °C, 32 °C and 39 °C, respectively. It is specified that higher values of bearing capacities were attained as the curing temperatures were raised.

The result that increasingly higher ratios of bearing capacity improvements were attained for increasing curing temperatures points out that high curing temperatures have a very significant effect on the occurrence of geopolymerization reactions. However, since it can be challenging and problematic to reach and sustain high curing temperatures in the field at the application stages, it is at least advised to implement such procedures in the summer months in order to optimize the improvements even further.

### 3.6. The Effect of Curing Durations

**Figure 20:**
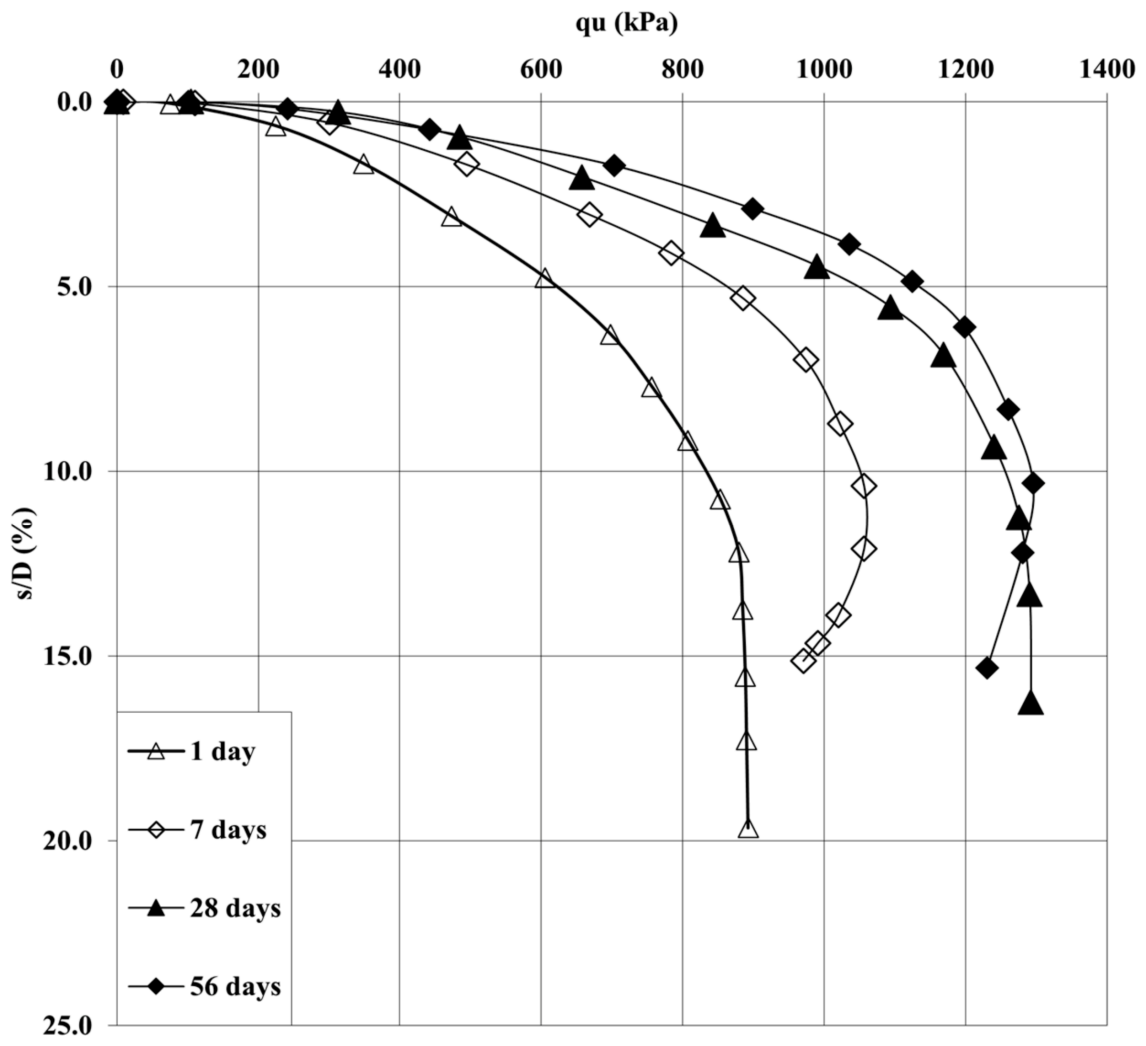
Load–displacement curves for various curing durations.

**Figure 21:**
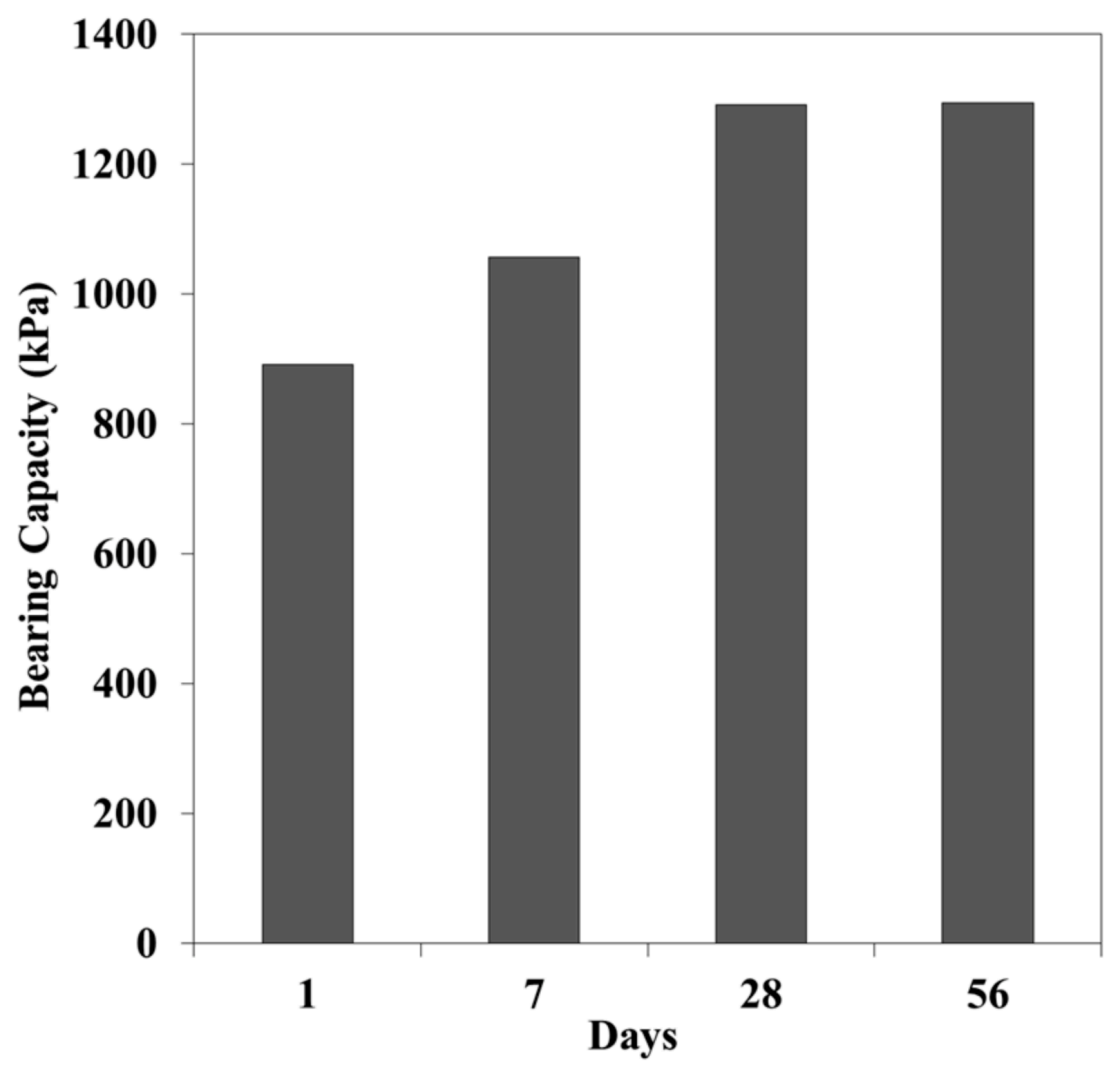
Bearing capacity values for various curing durations.

The mixtures prepared at optimum ratios and activated by the utilization of the most ideal activator conditions were subjected to curing durations of 1, 7, 28 and 56 days in order to analyze the effects of curing duration on the improvement ratios. The obtained results are presented in Figure 20 and Figure 21. According to the acquired results, the values of bearing capacities were recorded as 879.29 kPa, 1056.49 kPa, 1290.33 kPa and 1295.53 kPa in the sequel of 1-, 7-, 28- and 56-day periods, respectively. It was observed that the bearing capacities increased significantly as the curing durations were increased up to the end of day 28. Considerable improvements in the bearing capacity values were not encountered as the 28-day curing duration was exceeded. Thus, in soil improvement work implementing the utilization of geopolymer materials, waiting for a period of 28 days in advance of the initiation of any constructional activity upon such soil conditions is advised.

### 3.7. The Effect of Water Permeability

**Figure 22:**
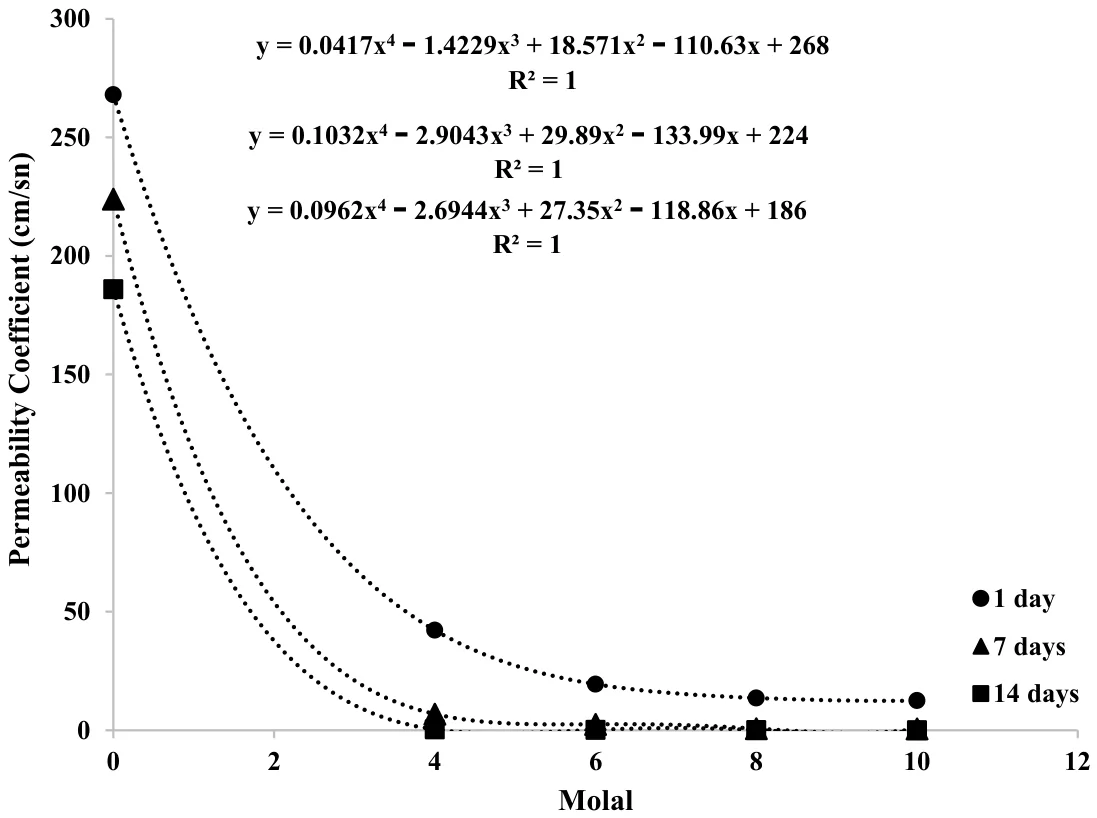
The effect of geopolymers on permeability.

The samples prepared at optimum mixture ratios and by means of the most ideal activator were experimented on after the completion of 1-, 7- and 14-day curing periods. The achieved results can be seen in Figure 22. In accordance with the results, reductions in the permeability values for molarities of 4, 6, 8 and 10 at the sequel of 1-, 7- and 14-day curing periods at ratios ranging in between 84.3–92.8%–94.9–95.3%, 97.0–98.9%–99.7–99.7%, 99.8–99.9%–100–100% were attained compared to the case where just clayey samples were used. Thus, it can be pointed out that significant improvements were achieved in the values of permeability by means of utilizing geopolymers in soil improvement procedures. It was considered that as the imperviousness of the soils increase, water retention capability and swelling-consolidation behavior for the soils in question are improved, as well.

### 3.8. Settlements with Respect to Time

**Figure 23:**
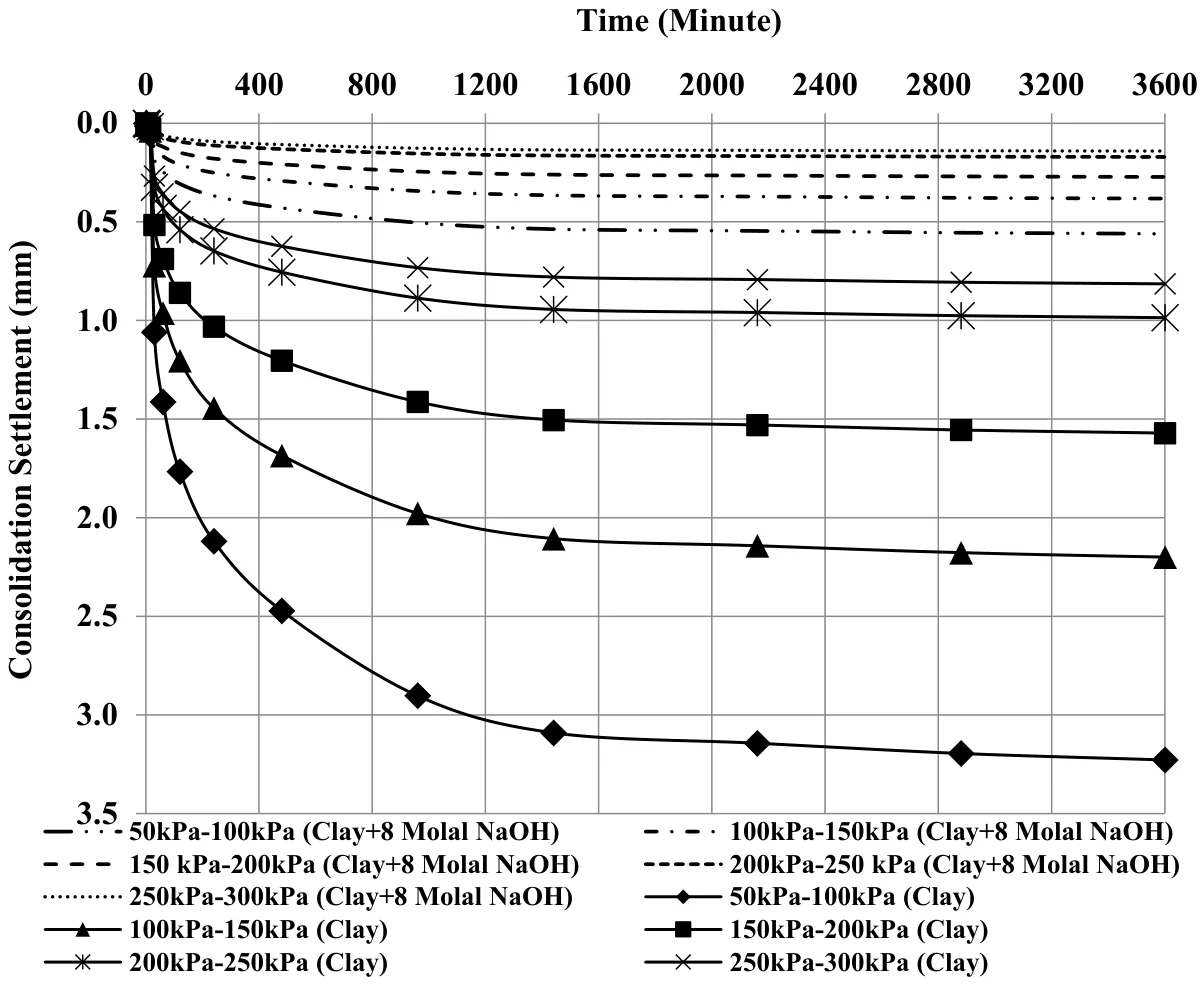
The correlation of consolidation settlements–time for the mixtures.

A series of experiments were performed in order to specify the values of consolidation settlements [60] taking place on the ground in regard to time under the influence of various loads exerted on the samples. In Figure 23 below, the acquired results of the conducted experiments are presented. Reduction ratios ranging between 5.76 and 5.78 times were attained in the values of consolidation settlements for the applied loading levels in comparison with the unimproved clay condition. Hence, it was expressed that CDW–geopolymer mixtures provided significant degrees of reductions in consolidation settlements and lead to increments in the bearing capacity values of the utilized clayey soil samples.

### 3.9. Comparison of Bearing Capacity Between Geopolymers and Cement

Traditional additives are generally used in the improvement of sand and clayey soil conditions. Within the context of a conducted study, the optimum ratio of cement was determined to be 10% in regard to the utilization of the mentioned material in soil improvement procedures [61,62]. Concordantly, additional large-scale-model laboratory experiments were carried out with the utilization of 10% cement in the mixtures of soil and construction demolition wastes prepared at optimum mixture ratios and cured for the periods of 1, 7, 28 and 56 days in order to perform a decent comparison between cement and geopolymers. As the results of the carried-out experiments were analyzed, the increment ratios acquired in the bearing capacity values for cement utilization at the end of the curing periods of 1, 7, 28 and 56 days were recorded as being just 1%, 3%, 4% and 4% more on average, respectively, compared to the case of geopolymers.

**Figure 24:**
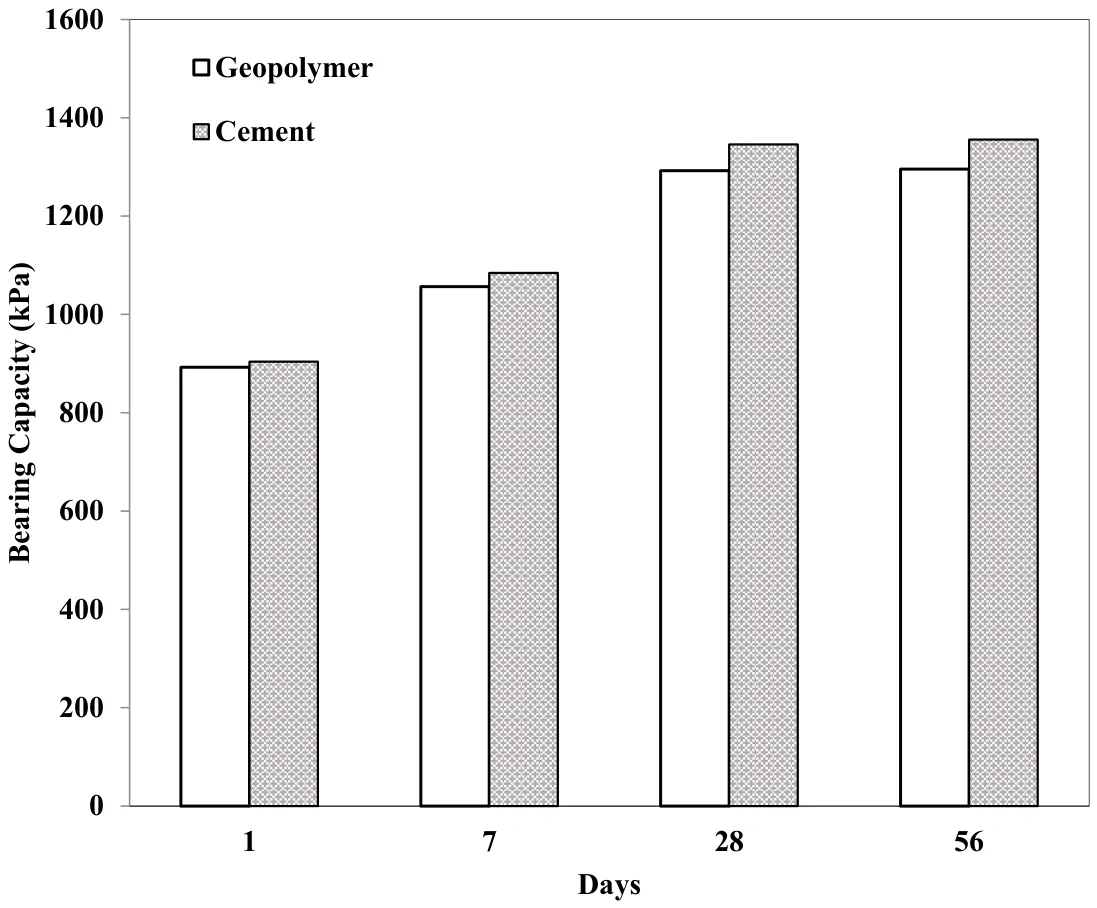
Comparison between geopolymers and cement.

The correlations obtained as a result of the performed experiments are presented in Figure 24 below. Consequently, geopolymers, in regard to enabling more economical solutions and providing almost the same degrees of improvements in the bearing capacity values of the utilized soil conditions as cement, were designated to be a considerable alternative to the currently implemented soil-strengthening additives.

## 4. Conclusions

The conducted study aimed to analyze several parameters in regard to the improvement of clayey soils by means of the utilization of the geopolymerization method. Eventually, the results given below were obtained.

- As a result of the large-scale model experiments performed in a laboratory environment, the optimum ratio of construction demolition wastes to be implemented in such soil conditions was determined to be 14%, whereas the value of optimum water content was specified to be 17.05%.
- In the cases where clayey soils were improved by means of mixing with construction demolition wastes and activating the wastes by using NaOH, KOH and CaO alkaline activators, it was specified that the optimum molarity values for the mentioned alkaline activators should be taken to be 8, 8 and 6 molal, respectively.
- Out of the implemented alkaline activators, NaOH was deduced to be the most ideal one in terms of leading to the occurrence of bearing capacity improvements.
- It was identified that the bearing capacity of the utilized soil samples increased as the curing temperatures were raised. This phenomenon points out the significance of temperature effects on the occurrence of optimum states for such applications. Nevertheless, reaching and providing high-temperature conditions throughout such applications is not feasible in terms of economy and applicability. Due to the mentioned reasons, it is advised to plan such improvement work in the summer months in order to procure the most ideal outcome.
- It was observed that the values of the bearing capacity increased as the curing durations were held longer up to the conclusion of day 28 in the sequel of which considerable improvements were not encountered. Therefore, a minimum waiting period of 28 days should suffice after the completion of such soil improvement work before any constructional work is started.
- Significant improvements were observed in the permeability ratios for all curing durations. Thus, it can be expressed that the attained improvement degrees in permeability ratios by means of implementing such procedures lead to enhanced water retention ability and swelling-settlement behavior for the treated soil conditions.
- Reductions of approximately 5.76 to 5.78 times were achieved in the consolidation settlement degrees for different loading levels for CDW–geopolymer-treated clayey soils.
- It was also ascertained that the utilization of cement, which is a very widely known and implemented traditional additive, provided only 1% to 4% more bearing capacity values in comparison to the case of geopolymers. The phenomena in which the ratios of bearing capacity improvements acquired for cement are close enough to those of geopolymers and geopolymer materials enable much more economical and environmentally friendly solutions for the related procedures and bring out the significance of implementing geopolymers as an alternative soil improvement additive amongst other strengthening materials.

## Figures and Tables

**Table 1:** Chemical analysis results for the clayey soil and construction demolition wastes.

Component (%)	CaO	SiO_2_	Al_2_O_3_	Fe_2_O_3_	MnO	MgO	Na_2_O	SO_3_	K_2_O	TiO_2_	P_2_O_5_
Clayey Soil	3.20	50.6	18.4	8.70	3.10	6.1	2.50	-	3.10	1.65	0.65
CDW	58.4	19.2	8.7	5.6	-	4.3	1.2	1.1	0.8	0.4	0.3

**Table 2:** Mixing ratio of CDW.

	Mixing Ratio (%)								
CDW	0	2	4	6	8	10	12	14	20

**Table 3:** Mixing ratio of alkali activator.

	Mixing Ratio (M)			
NaOH	4	6	8	10
KOH	4	6	8	10
CaO	4	6	8	10

## Data Availability

The original contributions presented in this study are included in the article. Further inquiries can be directed to the corresponding author.
